# Association of potentially inappropriate medications with rehospitalisation and death within three months in older patients: a systematic review and meta-analysis

**DOI:** 10.1007/s11096-025-02013-y

**Published:** 2025-09-25

**Authors:** Isabella Muzzarelli, Vera Isabel Neumeier, Michael Gagesch, Wiebke Rösler, Andrea Rahel Burch

**Affiliations:** 1 Hospital Pharmacy, University Hospital of Zurich, 8006 Zurich, Switzerland; 2 Institute of Pharmaceutical Sciences ETH, 8049 Zurich, Switzerland; 3 University Clinic of Aging Medicine, Zurich City Hospital, 8037 Zurich, Switzerland; 4 Department of Geriatric Medicine and Aging Research, University of Zurich, 8006 Zurich, Switzerland; 5 Department of Medical Oncology and Hematology, University Hospital of Zurich, 8091 Zurich, Switzerland

**Keywords:** Beers criteria, Geriatrics, Humans, Inappropriate prescribing

## Abstract

**Introduction:**

Potentially inappropriate medications (PIMs) are medications with an unfavourable risk–benefit profile for patients aged ≥ 65 years. Currently, several screening tools are available and are used in clinical practice to identify PIMs.

**Aim:**

Our objective was to systematically synthesize the available evidence on the association between potentially inappropriate medications (PIMs), as identified by various screening tools including STOPP/START and the Beers Criteria, and the outcomes of rehospitalization and mortality within three months in older patients.

**Method:**

Adhering to Cochrane standards, we conducted a systematic review and meta-analysis to investigate the outcomes of patients aged 65 years and older, comparing those with at least one PIM identified by any explicit screening tool to those without PIMs. Primary outcomes were readmission and/or death within three months. The databases of Embase, MEDLINE, and CENTRAL were searched for retrospective as well as prospective observational studies published between 1991 and May 17 2024. The risk of bias was assessed for all included studies.

**Results:**

In total, 1,642 studies were identified through the systematic search. Nine observational studies were included in this review. Our analysis revealed a statistically significant association between the overall presence of PIMs and rehospitalisation; odds ratio (OR) 1.47 [95% confidence interval (CI) 1.02 to 2.13, *p* = 0.045]. While stratification according to STOPP/START criteria yielded a statistically significant OR of 1.84 [CI 1.08 to 3.12, *p* = 0.024; n = 5], no statistically significant difference was seen according to Beers list [OR 1.25, CI 0.86 to 1.81, *p* = 0.235; n = 5]. Furthermore, no significant association was found between PIMs and mortality in either the overall analysis or the stratification according to the Beers and STOPP/START criteria. The risk of bias in the included studies was moderate to serious, and the certainty of evidence was very low.

**Conclusion:**

The use of PIM screening tools during medication evaluations may reduce the risk of rehospitalisation and potentially lower mortality in older patients. However, further studies are warranted to confirm the association with adverse outcomes.

**Supplementary Information:**

The online version contains supplementary material available at 10.1007/s11096-025-02013-y.

## Impact statements

- Potentially inappropriate medications (PIMs) lead to higher odds of rehospitalisation of patients aged 65 years and older within three months.
- The association between PIMs and mortality is unclear due to lack of reported data.
- PIM screening tools define PIMs differently and therefore, they associate differently with adverse outcomes in older patients.

## Introduction

Potentially inappropriate medications (PIMs) are defined as medications that present an un- favourable risk-benefit profile for patients 65 years of age and older [1]. The older population is particularly vulnerable due to age-associated changes in pharmacokinetics, multiple coexisting chronic diseases (i.e., multimorbidity), and a high prevalence of geriatric syndromes and functional impairments [2]. As a result, older patients are often prescribed multiple medications, leading to polypharmacotherapy, which is commonly defined as the concurrent use of five or more different medications [3]. This increases the likelihood of at least one PIM being prescribed [4]. Optimising medication in this patient group presents an opportunity for physicians and pharmacists to potentially reduce adverse drug events (ADEs) [5]. Pharmacist-supervised medication reviews have proven beneficial in preventing ADEs, PIMs, and other medication-related problems in the inpatient setting [5–8] and can reduce the prevalence of PIM in this cohort [9].

Several screening tools have been developed to identify PIMs to improve overall prescribing practices in older adults. The two most well-known tools are the Beers criteria (commonly known as the “Beers list”) and the STOPP/START criteria [10, 11]. The Beers criteria list was the first PIM screening tool overall [12], categorising PIMs into medications to avoid, medications to avoid under certain conditions and medications to use with caution. Beers also gives suggestions for alternative approaches and provides grades of evidence [10].

The STOPP/START criteria consist of two parts: The first, STOPP, defines drug classes and selected medications as PIMs dependent on a wide range of conditions such as indications or comorbidities. The goal of the second part, START, is to alert physicians to medications particularly appropriate for older patients [11].

Another tool often used in German-speaking countries is the PRISCUS-List. It is a German-specific compilation of PIMs for older adults. The list includes 83 drugs across 18 therapeutic classes. Each listed medication is accompanied by recommendations for clinical alternatives and practical advice for safer prescribing [13].

The association of PIMs with adverse outcomes in older adults has been described before in several published articles [14–16]. Assessed outcomes, exposures, PIM tool applications, population, and study periods vary greatly from study to study.

Several reviews, including one by Hyttinen et al. [17] (39 studies included), have reported significant associations between PIMs and increased healthcare use, such as hospitalisations and ED visits [16]. Mekonnen et al. [18] (63 studies included) found that PIMs during hospitalisation were linked to outcomes such as ADEs, falls, and higher healthcare costs, although no significant association with mortality or readmissions was observed after adjusting for confounders. A previously published meta-analysis by Xing et al. [19] (33 studies included) reported an association between PIMs and the risk of unplanned hospitalisation as well as ADEs, although the time frame for these outcomes was not specified. Similarly, Liew et al. [20] (8 studies included) found higher risk ratios for emergency room visits, ADEs, and hospitalisations associated with PIMs in their meta-analysis.

In contrast, a 2017 meta-analysis by Muhlack et al. [21] (13 studies included) suggested a potential link between PIMs and increased mortality. This finding aligns with a systematic review conducted by Mohamed et al. [4], which included studies involving cancer patients (2020, 47 studies included).

These discrepancies may, in part, be due to differences in care settings, as also highlighted by the study by Jano et al. [22] (18 studies included). Additionally, the influence of methodological choices on reported outcomes is also evident. All of the mentioned reviews included studies investigating the population “older adults”, and did not separately analyze them.

Moreover, evidence regarding the timing of adverse events remains diverse limited. A follow-up period of three months is commonly employed in studies evaluating ADEs for several reasons: First, many adverse effects associated with PIMs tend to manifest within weeks to a few months after initiation or modification of therapy, making this timeframe clinically relevant for capturing early drug-related harms [23–25]. Second, limiting observation window reduces confounding by other long-term health changes or interventions that may occur over extended periods and complicate causal interference [26]. Third, the three-month period balances the need for sufficient follow-up to observe outcomes with practical considerations in study design, such as minimising loss to follow-up and ensuring timely data collection [27]. Finally, this timeframe aligns with clinical practice, where medication reviews and interventions often occur quarterly, facilitating translation of research findings into patient care [28].

For example, Schwab et al. [29] concluded that the association between PIM use and hospitalisation within 30 days remains inconclusive. This illustrates the need for more evidence of the described relationship within specified time frames, since especially larger time periods increase the likelihood of an undesirable effect or adverse event leading to rehospitalization or even death.

### Aim

The aim of this study was to systematically investigate the current evidence base for PIMs, focusing on the rates of unplanned rehospitalisation and mortality within a three- month time period, potentially linked to PIMs in older adults.

## Method

### Eligibility criteria

In accordance with the Cochrane Handbook of Systematic Reviews, we designed a study protocol adhering to the PRISMA-P checklist to ensure transparency and comprehensiveness [30, 31]. The study protocol was registered in an international prospective register of systematic reviews (PROSPERO) (CRD42024525873). Changes made to the protocol after registration are explained in electronic supplementary material S1.

We established inclusion and exclusion criteria for publications as follows: The study designs we would have included were observational studies and randomised controlled trials (RCTs). The publication dates ranged between 01.01.1991 and 17.05.2024, since the Beers list, the first tool to systematically screen for PIMs, was published in 1991 [12]. All other study designs as well as publication languages other than English or German were excluded. The included study populations were patients aged ≥ 65 years of all genders and geographical regions. Interventions were defined as PIMs detected by any screening tool and consequently, comparisons were defined as no PIMs detected by the same screening tool. Finally, included outcomes were unplanned rehospitalisations and/or death within three months. The outcome “unplanned rehospitalisations” included any kind of unplanned readmission to a stationary setting, such as acute readmission to hospital after discharge or during follow-up, ED presentations, all-cause ED visits, and ED room revisits and readmissions.

### Search strategy and databases

We searched databases EMBASE [32], MEDLINE [33], and CENTRAL [34] comprehensively and systematically for studies (see electronic supplementary material S2). No filters were applied. The search was conducted on April 5 2024 by the first author. An update search was conducted on May 17 2024 before the start of data synthesis by the same author.

### Study selection

Duplicate removal was performed manually by comparing titles, publication years, and authors, and checking the digital object identifier (DOI) when available. Two independent researchers screened titles and abstracts (VN, IM). We then conducted full-text screening (VN, IM). Potential disagreements were resolved by a third researcher (AB, VN, IM).

### Data extraction and analysis

Two independent researchers extracted and stored the data from the included studies in an Excel table (version 2016) [35]. In case of missing data, authors were contacted via email. After comparing the extraction, we resolved all differences by consensus and, if necessary, involved a third independent researcher.

### Risk of bias assessment

We assessed the risk of bias (RoB) of the included studies using the risk of bias in non-randomised studies of interventions (ROBINS-I) tool [36]. This tool evaluates the biases in studies due to confounding, participant selection, intervention classification, deviations from intended interventions, missing data, outcome measurements, and the selection of reported results. This assessment is conducted separately for each study and each outcome.

### Data synthesis

We used the R software environment (version 4.4.0) and the metafor package for data processing, model parameter estimation, and presentation [37, 38]. For each of the two defined outcomes (rehospitalisation and mortality), we performed a separate meta-analysis using a random-effects model with the rma() function from the metafor package. The between-study variance (τ^2^) was estimated using the Restricted Maximum Likelihood (REML) method, which is the default in metafor. No additional statistical adjustments (e.g., Hartung–Knapp, small-sample corrections, or robust variance estimation) were applied.

The measures of effect for the outcomes were reported as odds ratios (OR) with a 95% confidence interval (CI). The calculations of the variables are listed in the electronic supplementary material S6.

All analyses and forest plots were created using the metafor package, which also generated the pooled ORs and corresponding 95% CIs. An OR >1.00 suggested higher odds of rehospitalisation with the presence of PIMs. Statistical significance was achieved if the 95% CI (i.e., *p*-value ≤0.05) did not include the value 1.00. The forest plots for both outcomes were stratified according to PIM tool application.

To avoid falsification, we included only a single OR from each study in the analysis. Inclusion of several ORs for the application of different PIM tools could have resulted in those studies being weighed too heavily. When multiple ORs were reported, we chose the highest OR for our analysis.

For studies that only provided the OR and 95% CI, as well as the total number of patients but not the individual numbers of patients in the intervention and comparison groups as well as the numbers of events in each group (e.g., individual cell counts (a, b, c, d)), we used numerical optimisation via the **nloptr** package in R [39]. Specifically, we applied the NLOPT_LN_SBPLX algorithm (a derivative-free, bound-constrained simplex method) to minimise the squared difference between calculated and reported ORs and CIs under known marginal constraints.

### Heterogeneity assessment

We carried out the *χ*^2^ test and calculated *τ*^2^, the Higgins *I*^2^ statistic, and prediction intervals (PI) to both qualitatively and quantitatively assess heterogeneity.

### Publication bias

Funnel plots were inspected visually to assess publication bias. A minimum of 10 studies needs to be included to detect asymmetry [30].

### Sensitivity analysis

We conducted a sensitivity analysis for both outcomes by only including studies with moderate RoB and excluding studies with serious RoB.

### GRADE assessment

The quality of evidence of both outcomes was evaluated using the Grading of Recommendations Assessment, Development, and Evaluation (GRADE) approach [30, 40].

## Results

### Study selection

**Fig. 1:**
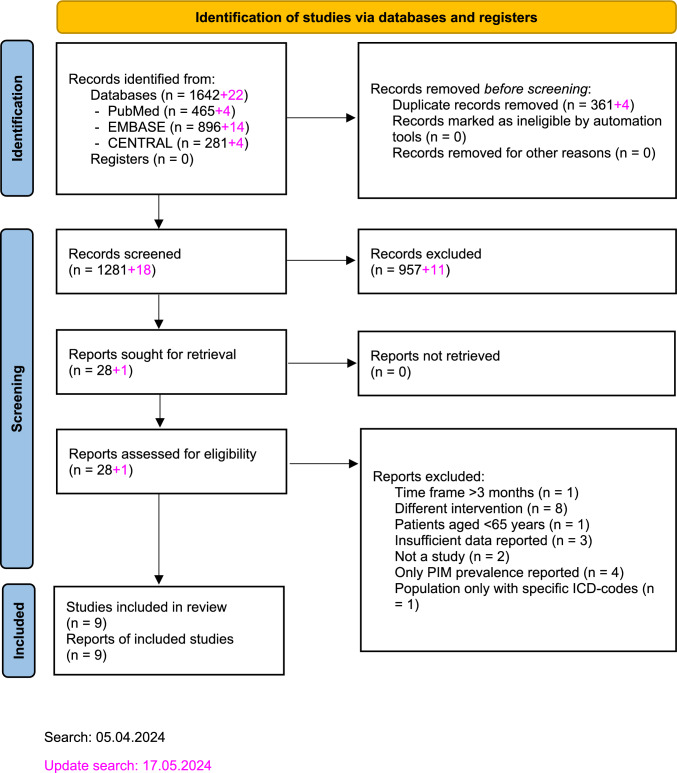
Flow diagram of study screening and selection

In total, 1,642 records were identified, 361 of which were removed as duplicates. During the re-run, an additional 22 records were found and screened. The entire screening process resulted in nine studies being included in our meta-analysis. The literature selection process is depicted in Fig. 1. The reasons for exclusions during full-text screening are explained in the electronic supplementary material S3.

### Characteristics of included articles

Nine studies were included in the systematic review, reporting on either rehospitalisation and mortality within three months [41–44] or on both outcomes [45–49]. By design, four were cohort studies [41, 44–46], two other observational studies [43, 47], one was a survey [49], one a cross-sectional study [48], and one a nested case-control study [42]. Four studies were conducted in Europe (three in Italy) [43, 45, 48, 49], two in Australia [46, 47], two in North America [41, 42], and one in Taiwan [44].

Only one study, which is also the only study to report on mortality exclusively, assessed patients in outpatient treatment [42], whereas the remaining studies included patients in various hospital inpatient settings [41, 43–49].

The sample sizes of the study population in the studies varied between 232 [46] and 174,275 patients [41]. All patients were aged 65 years and older, while the mean ages ranged from 76.7 [42] to 83.0 years [49].

All but three studies used a version of the Beers list to screen for PIMs [41–45, 48], whereas STOPP/START was reported in four publications [41, 43, 46, 47]. In one publication medication was screened using a different tool from either the Beers list or STOPP/START criteria [49].

The study periods ranged from five months [46] up to seven years [45].

Table 1 presents the study characteristics of the nine included studies. More details can be gathered from the summary of findings see electronic supplementary material S5.

**Table 1:** Study characteristics of included studies

References	Location (Country), Study period	Study design	Setting	Sample size (% female)	Mean age ± SD	Population included	Screening tool (version)	Outcome description
Mekonnen [46]	Australia/ 03.2021–08.2021	Retrospective cohort study (followed by phone interviews)	General medicine services	232 (51.7%)	80.0 ± 7.9	≥65 years, with hospital stay ≥ 1 day, consecutively discharged alive to usual residency	STOPP (v2)	Hospital readmissions, ED presentations, and composite readmissions and/or ED presentations within 3-months of index hos- pitalisation
Mekonnen [47]	Australia/ 10.2017–03.2020	Prospective observational study (followed by phone interviews)	Hospital for geriatric rehabilitation	1890 (56.3%)	82.6 ± 8.1	From RESORT cohort, admitted to ge- riatric rehabilitation, informed consent	STOPP (v2)	Hospital readmissions (i.e., unplanned acute readmission after discharge) and mortality at 3 or 12 months post discharge
Fabbietti [43]	Italy/ 01.2013–12.2013	Prospective observational study	Acute care wards of ge- riatric medicine	647 (49.0%)	80.1 ± 6.9	≥65 years admitted to any of the wards	Beers (2015), STOPP (v2)	Any admission during 3- month follow up after discharge
Renom-Guiteras [49]	8 European countries*/ 11.2010–04.2012	Prospective survey	Institutional long-term care or home care	2004 (67.5%)	83.0 ± 6.6	≥65 years with diagnosis of dementia, 24 or lower points in standardised mini mental state examination, with an informal caregiver	EU(7)-PIM list	Mortality, fall-related injury or hospitalization after 3 months
DeVincentis [45]	Italy/ 01.2010–12.2016	Prospective cohort study	Medical wards	2631 (51.4%)	Median (IQR): 79.6 ± 12	≥65 years who were discharged at home, discharged at home from REPOSI cohort	Beers (2019)	Survival, occurrence of rehospitalisation and functional status decline at 3 months from discharge
Brown [41]	USA/ 01.2006–12.2009	Retrospective cohort study	Managed care organisations within US	174275 (54.3%)	n.a	≥65 years, ≥ 9 months of continuous medical and pharmacy coverage with 6 months pre-index period and 3 months follow-up	Beers (2003), Beers (2012), STOPP (v1)	ADEs, all-cause ED visits, or all-cause hospitalisations in following month after PIM exposure
Pasina [48]	Italy/ 01.2010–12.2010	Prospective Cross-sectional	Internal Medicine and geriatric wards	844 (51.2%)	78.8 ± 7.4	≥65 years, signed informed consent, first 10 patients of each month from REPOSI cohort	Beers (2003), Beers (2012)	Prevalence of PIMs, risk of AEs from discharge to follow-up date (3 months), rehospitalisation and all-cause mortality at follow-up (3 months)
Liang [44]	04.2017–12.2017	Retrospective cohort study	Internal medicine ward	2671 (39.6%)	77.2 ± 8.4	≥65 years admitted to internal medicine ward for the first time in study period, no hospice or limited life expectancy due to terminal illness, not in ICU	Beers (2015)	ED room revisits and readmissions at 1, 3, and 6 months post discharge
D’Aiuto [42]	Canada/ 01.2011–05.2017	Retrospective nested Case–control study (in Underlying cohort of opioid users)	Ambulatory	200 (58.5%)	76.7	≥65 years with opioid prescription claim During follow-up period (1, 1.5, 2, 3, and 4 months) and none before and without malignant tumour diagnosis	Beers (2019)	All-cause mortality within 90 days

*AE* Adverse event; *ADE* Adverse drug event; *ED* Emergency department; *ICU* Intensive Care Unit; *IQR* Interquartile range; *PIM* Potentially inappropriate medication; *SD* Standard deviation

*England, Estonia, Finland, France, Germany, the Netherlands, Spain and Sweden

### Risk of bias assessment

The RoB assessment (ROBINS-I tool) classified the RoB of all studies as either moderate or serious (Table 2). Out of the eight studies with the outcome rehospitalisation within three months, Brown et al. [41], DeVincentis et al. [45], and Liang et al. [44] were classified as moderate. The RoB of DeVincentis et al. [45] and D’Aiuto et al. [42] out of the six studies with the outcome mortality within three months were moderate.

**Table 2:** Overview of the overall risk of bias classifications of all studies by outcome by application of ROBINS-I tool

Study	Primary Outcome: Rehospitalisation within 3 months	Secondary outcome: Mortality within 3 months
Brown [41]	Moderate risk	NA
D’Aiuto [42]	NA	Moderate risk
DeVincentis [45]	Moderate risk	Moderate risk
Fabbietti [43]	Serious risk	NA
Liang [44]	Moderate risk	NA
Mekkonnen [46]	Serious risk	Serious risk
Mekkonnen [47]	Serious risk	Serious risk
Pasina [48]	Serious risk	Serious risk
Renom-Guiteras [49]	Serious risk	Serious risk

### Characteristics of selected studies

In total, nine studies for inclusion in the meta-analysis were identified (see Table 1). While some studies only reported on rehospitalisation within three months, others reported exclusively on mortality within the same timeframe.

The studies by Fabbietti et al. [43], DeVincentis et al. [45], Brown et al. [41], and Pasina et al. [48] analysed their cohort several times with different PIM tools or different versions of PIM tools. As explained in the Methods sec- tion, only one OR from each study was included in the analysis. Forest plots of the analysis with the ORs of these studies that were not included in the main analysis can be found in the electronic supplementary material S7. DeVincentis et al. [45] and Pasina et al. [48] both analysed patients of the same cohort (the REPOSI cohort). DeVincentis et al. [45] analysed a subset of patients treated in the medical wards between 2010 and 2016, whereas Pasina et al. [48] used the data of patients in the wards exclusively from the year 2010. Therefore, the overlap of included patients was low enough to include both effect measures in the meta-analysis without the danger of weighing the same population twice.

The studies Brown et al. [41] and Renom-Guiteras et al. [49] did not report the individual numbers of rehospitalised patients with PIMs but only the OR and 95% CI as well as the total numbers of outcomes. Therefore, the numbers of patients in each group were approximated.

### Association of PIM with rehospitalisation

**Fig. 2:**
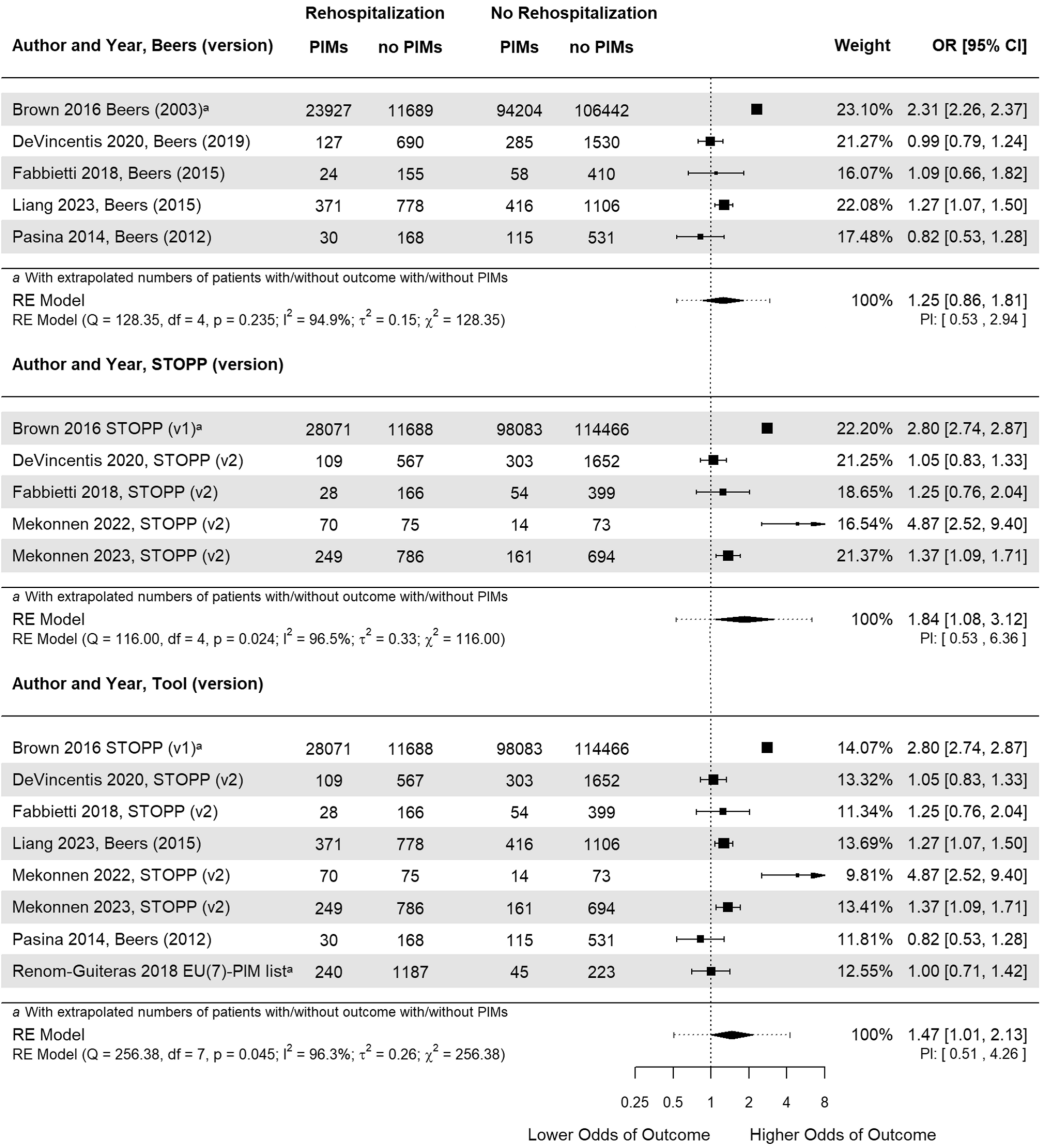
Forest plot of the association between potentially inappropriate medications (PIMs) and rehospitalisation within three months. The width of the diamond shape of the overall odds ratio (OR) in- dicates the 95% confidence interval (CI). The dotted line represents the prediction interval (PI). *CI* Confidence Interval; *OR* Odds Ratio; *PIM* Potentially inappropriate medication; *RE* Random effect; *STOPP* Screening tool of older people’s prescriptions

We included eight studies on the association of PIMs with rehospitalisation within three months in older patients [41, 43–49]. The analysis of all estimates, regardless of PIM criteria, resulted in a statistically significant pooled OR estimate of 1.47 [CI 1.02 to 2.13, *p* = 0.045] (Fig. 2). Thus, the odds of rehospitalisation were higher if PIMs were prescribed. Forest plots of the analysis with other reported ORs of the studies reported results on several tools can be found in the electronic supplementary material S7. The Q-test revealed considerable heterogeneity as indicated by the *χ*^2^ and *τ*^2^ values as well as the Higgins *I*^2^ statistic.

The association of at least one PIM according to STOPP/START criteria with rehospitalisation within three months was deemed statistically significant, whereas this cannot be said for Beers criteria. Stratification by PIM tool, i.e., Beers and STOPP/START regardless of version, yielded pooled OR estimates of 1.25 [CI 0.86 to 1.81, *p* = 0.235] and 1.84 [CI 1.08 to 3.12, *p* = 0.024], respectively (Fig. 2). The values *χ*^2^ and *τ*^2^, the Q-test, and *I*^2^ suggest considerable hetero- geneity among the included studies in both subgroups.

### Association of PIM with mortality

**Fig. 3:**
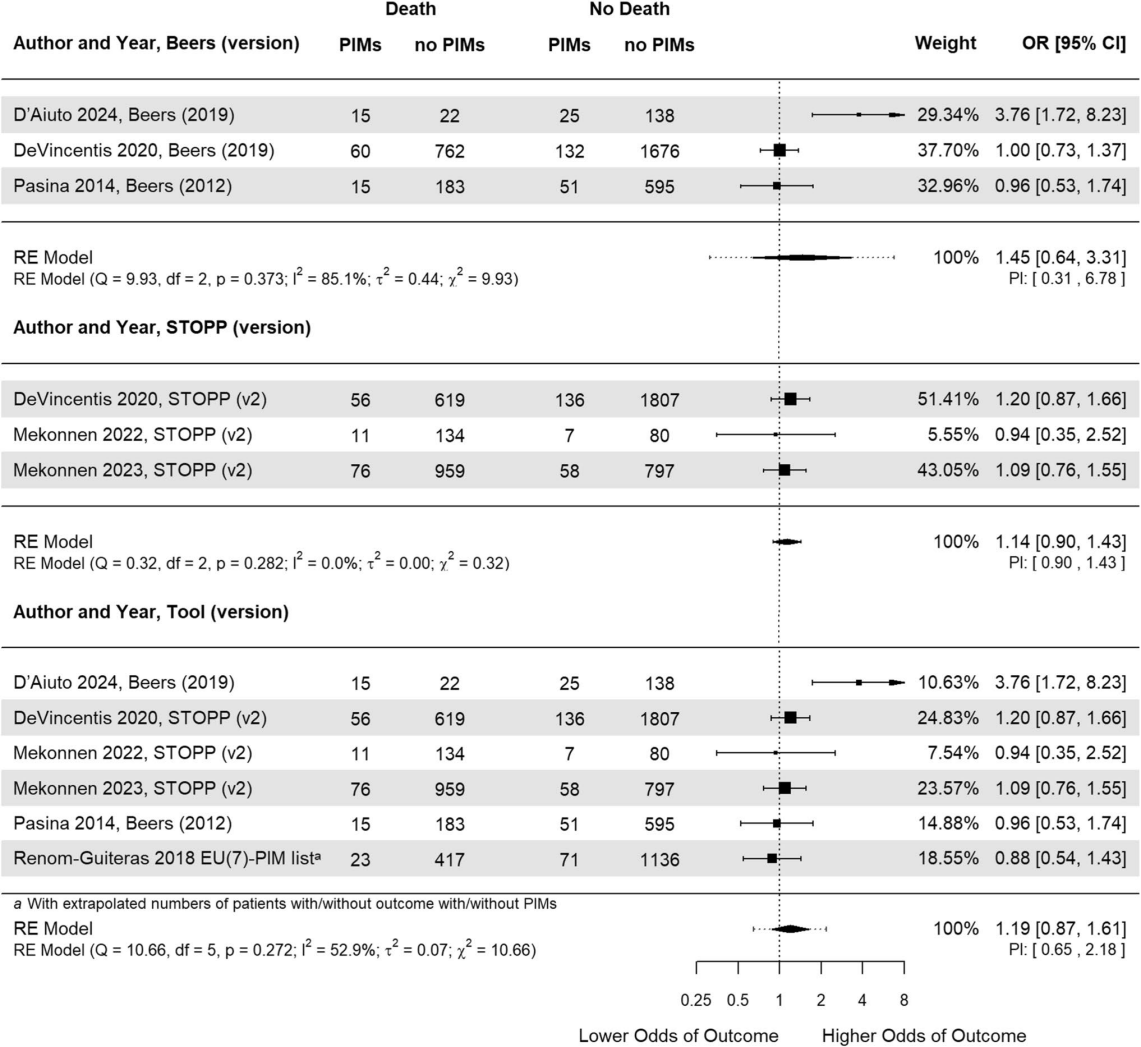
Forest plot of the association between Potentially Inappropriate Medications (PIMs) and mortality within three months. The width of the diamond shape of the overall odds ratio (OR) indicates the 95% confidence interval (CI). The dotted line represents the Prediction Interval (PI). CI, Confidence Interval; OR, Odds Ratio; PIM, Potentially Inappropriate Medication; RE, Random Effect; STOPP, Screening Tool of Older People’s Prescriptions

We identified a total of six studies reporting on the association between PIMs and mortality within three months in older patients [42, 45–49]. The pooled OR estimate of 1.19 [CI 0.87 to 1.61, *p* = 0.272] did not show a statistically significant association between PIMs and mortality within three months (Fig. 3). The *χ*^2^, *τ* ^2^, Q-test, and the Higgins *I*^2^ statistic suggest some heterogeneity. Visually, the forest plot looks irregular due to the effect estimate presented by D’Aiuto et al. [34].

Stratification by Beers and STOPP/START (regardless of version) did not show statistical significance either with pooled OR estimates of 1.45 [CI 0.65 to 3.31, *p* = 0.373] and 1.14 [CI 0.90 to 1.43, *p* = 0.282], respectively. The model for STOPP/START yields an Higgins *I*^2^ statistic of 0%, due to the value for degrees of freedom (df) being higher than the Q-value.

## Publication bias

Only nine studies could be included in the meta-analysis. The funnel plots generated for the different outcomes did not all show apparent asymmetry or a distinct pattern suggesting substantial publication bias. However, given the limited number of studies and heterogeneity, we acknowledge that the absence of apparent asymmetry does not definitively rule out the possilibity of publication bias. The funnel plots for all forest plots can be found in electronic supplementary material S9.

### Sensitivity analysis

A sensitivity analysis was performed to gauge the robustness of the effect estimates by excluding studies with a high RoB. The sensitivity analysis for either outcome did not yield statistical significance (see electronic supplementary material S8). The heterogeneity did not differ much.

### GRADE assessment

For all outcomes and subgroups, we graded the certainty of evidence as “very low” (Table 3).

**Table 3:** GRADE evidence profile of PIMs in geriatric patients

Study characteristics		Quality assessment						Number of patients		Effect	GRADE
PIM list	Outcome	#, Study type	RoB	Imprecision	Inconsistency	Indirectness	Publication Bias	Intervention: Outcome/ total	Comparator: Outcome/ total	OR (95% CI)	GRADE
Beers	Rehospitalisation	5, nRCT	Serious	Serious	Serious	Serious	Undetected	28,080/43987	88,195/192505	1.25 (0.87, 1.79)	Very low
Beers	Death	3, nRCT	Serious	Serious	Serious	Serious	Undetected	90/1057	208/2617	1.45 (0.64, 3.31)	Very low
STOPP	Rehospitalisation	5, nRCT	Serious	Not serious	Serious	Serious	Undetected	28,527/41809	98,615/215899	1.84 (1.08, 3.12)	Very low
STOPP	Death	3, nRCT	Serious	Serious	Not serious	Serious	Undetected	143/1855	201/2885	1.14 (0.90, 1.43)	Very low
EU(7)-PIM list	Rehospitalisation	1, nRTC	Serious	Serious	n.a.^1^	Serious	Undetected	240/1427^*a*^	45/268^*a*^	1.00 (0.70, 1.41)	Very low
EU(7)-PIM list	Death	1, nRTC	Serious	Serious	n.a.^1^	Serious	Undetected	23/437^*a*^	71/1207^*a*^	0.86 (0.53, 1.40)	Very low

*CI* Confidence interval; *GRADE* Grading of recommendations assessment, development, and evaluation; *nRCT* Non-randomised controlled study; *OR* Odds ratio; *PIM* Potentially inappropriate medication; *RoB* Risk of bias

^1^Single study; Inconsistency is not applicable

^a^Numbers are extrapolated from OR, CI and total numbers of outcomes

## Discussion

### Statement of key findings

Our systematic review and meta-analysis on the association of PIMs with rehospitalization and mortality in patients 65 years and older demonstrates a statistically significantly higher OR for rehospitalisation within three months in the presence of one or more prescribed PIMs. This association also applied to the subgroup analysis of only the STOPP/START criteria, but not for PIMs defined by the Beers criteria. No significant association between one or more PIMs of any tool and mortality within three months could be found.

### Strengths and weaknesses

Our study has several strengths. First, we adhered closely to the Cochrane Handbook of Systematic Reviews and conducted our methods and analysis in a well-planned manner [30]. Our systematic review was conducted and reported in accordance with PRISMA-P standards [31]. To our knowledge, this is the first meta-analysis to characterise the association between PIMs and rehospitalisation in older patients within three months.

Some limitations need to be acknowledged. First, our sensitivity analyses including only studies with moderate RoB did not yield statistically significant ORs for any outcome. This suggests that the significant result may be driven, at least in part, by studies with a higher risk of bias. In other words, the apparent effect might be overestimated or less reliable due to the influence of less rigorous studies.

Secondly, we used the highest ORs from studies reporting multiple ORs (due to various PIM tools) in our main anal- yses, while analyses with lower ORs failed to produce a significant effect. For some studies, crude 2×2 data were reconstructed from summary statistics, which may introduce estimation uncertainty. Additionally, although the pooled ratio suggests a significant association, the crossing of the predictive interval with the value 1 indicates that future studies under similar conditions may find no association or even an opposite effect. This reflects some heterogeneity among the study results, suggesting that the evidence may not be equally robust across all contexts. Furthermore, we did not apply the Hartung-Knapp-Sidik-Jonkman (HKSJ) adjustment to the CIs in our random-effects meta-analyses. While HKSJ is often recommended with few studies and high heterogeneity, we chose not to use it for two reasons. First, HKSJ can produce overly conservative estimates with very wide confidence intervals, reducing interpretability and power in small samples [50, 51]. Second, we followed the approach of Xing et al. [19], who conducted a similar meta-analysis and did not use the HKSJ adjustment. While HKSJ might have been statistically preferable given the heterogeneity of the data, we prioritised consistency with prior work and standard methods in the metafor package. Finally, the GRADE assessment was “very low” for all outcomes. This low certainty was mainly driven by the observational nature of the included studies, which inherently carry a higher risk of bias and do not allow for causal inference. Many studies were also limited by small sample sizes, reducing precision and statistical power. These limitations reduced the statistical power, robustness, and credibility of our findings. This was greatly influenced by the quality, statistical power, and robustness of the included studies, all of which were observational studies that precluded the possibility of establishing causality. Furthermore, many studies may lack precision in their estimates due to the small number of people included. The availability of prescribed drugs and therefore possible PIMs differ greatly between countries, which further complicates the comparison between Australia, North America, Taiwan, and Europe. Demographics and availability of data and health- care may also vary greatly between countries. In addition, most researchers did not have or did not have much information on adherence to medications as well as on non-prescription drugs.

### Interpretation

Our findings of the association between PIMs and rehospitalisation in older patients appear in line with the current literature for follow-up time periods of 30 days, six months, and 12 months [52–55]. Some publications, however, report no significant association. Notably, a systematic review by Schwab et al. [29] was not able to quantitatively describe the association between PIMs and readmission within 30 days. However, a meta-analysis by Xing et al. [19] showed a significant association of PIMs of either Beers or STOPP/START with rehospitalisation regardless of the time frame. In the stratification according to both PIM tools separately, this association was only significant for PIMs according to the Beers criteria, not STOPP/START, contrary to the results of the stratification in our meta-analysis.

The STOPP/START lists medications that are potentially inappropriate and should generally be stopped in older adults in Europe. In contrast to that, the Beers criteria emphasize medications to avoid, dose adjustments, and drug–disease interactions primarily in the US healthcare context. They focus mainly on medications to avoid, less on omissions or necessary medications to start. The PRISCUS does have a similar approach to make suggestions for change medications, rather than omissions in the German-speaking, European population. These differences in approaches how to handle inappropriate medications, but also the population for which they were developed can have an influence on effects when investigating outcomes such as rehospitalization or mortality.

The fact that no association between PIMs and mortality within three months could be made based on our results is consistent with several findings in literature with different defined time frames [55–58], including the meta-analysis conducted by Xing et al. [19]. However, there are publications reporting an association for the prescription of PIMs with higher odds of mortality within time frames of 30 days and six months [53, 54] or even six to 12 months in patients with ovarian cancer [59]. These more inconsistent results might be attributable to the fact that death is generally a rare event. In an aged and multimorbid population, death could be related more to co-morbidities and age rather than exclusively to PIMs.

### Further research

Further research, especially RCTs, is needed to establish causal relationships between PIMs and unplanned rehospitalisation and death. Additionally, the application of newer versions of PIM screening tools as well as lesser-known tools could provide more information on the applicability and predictive value regarding adverse outcomes. The applicability and generalizability of these findings should be carefully considered. Differences in healthcare systems, prescribing practices, and patient populations may influence how PIM tools perform in various settings. Large-scale studies with more extensive and diverse populations are essential to enhance the generalisability of results and to better under- stand the long-term impact of PIM use on clinical outcomes.

## Conclusion

Our findings support the use of PIM tools, especially the STOPP/START criteria, in medication re- views in order to help reduce the risk of rehospitalisation and potentially even mortality in patients aged 65 and older. A key approach to enhancing drug safety is for physicians to account for PIMs when prescribing to older adults. However, further research is needed to broaden the evidence base for the association between PIMs and adverse outcomes across different PIM tools and time frames.

## Supplementary Information

Below is the link to the electronic supplementary material.

Supplementary file1 (DOCX 2977 kb)

## Data Availability

Data are available upon request from the corresponding author.
