# Supplementary material for: Association of potentially inappropriate medications with rehospitalisation and death within three months in older patients: a systematic review and meta-analysis
Source: Int J Clin Pharm. 2025 Sep 25;48(2):350–63. doi: 10.1007/s11096-025-02013-y (PMC12992462; doi:10.1007/s11096-025-02013-y)
Supplement: Supplementary file 1 — Supplementary file1 (DOCX 2977 kb) [file 11096_2025_2013_MOESM1_ESM.docx]

# Supplementary Material

S1 Differences from the Protocol and the Final Review S2 Search Strategies

S3 Reasons for Exclusion of Articles in Full Text Review S4 Risk of Bias Judgement

S5 Summary of Findings S6 Calculation of Variables

S7 Forest Plots of Additional Analyses S8 Sensitivity Analysis

S9 Funnel Plots for Publication Bias Assessment

## S1- Differences from the Protocol and the Final Review

In order for the systematic review to match with the population of the observational study with the MOH clinic, the initial plan was to include studies with study populations of patients aged 70 and older[.[45]](#_bookmark50) However, during preliminary searches we discovered that the definition of ”older people”, ”geriatric people”, and other terminology much more commonly refers to people aged 65 and older rather than 70 years and older.

Population, intervention, comparison, outcome (PICO) assessment: In the protocol, we planned to compare the ORs if the Beers criteria, STOPP criteria, and the Priscus list are applied (inter- vention) in rates of rehospitalisation of patients due to PIMs (outcome) vsd the rehospitalisation rates if no tool is applied (comparison). The intervention and comparison of the PICO scheme had to be reassessed due to a lack of literature found in preliminary searches. We changed the intervention to ”PIMs detected by application of screening tool”, and the comparison to ”no PIMs detected by application of screening tool”. Furthermore, an additional primary outcome ”death” was included, whereas the outcome ”Number of PIMs detected” was omitted because it did no longer comply with the new intervention and comparison.

The additional subgroups analyses regarding the cumulative effects of only Beers criteria respectively only STOPP criteria application were added only after registration of the protocol as well.

## S2- Search Strategies

**First Search 05.04.2024**

### PubMed:


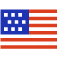
An official website of the United States government Here's how you know

**PubMed Advanced Search Builder**


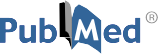


Add terms to the query box

All Fields

Enter a search term

ADD Show Index

Query box

Enter / edit your search query here

Search

**History and Search Details**

| Search  #4 | Actions  … | Details | Query  Search: ((((geriatr*) OR (geriatry) OR (geriatric) OR (older patient) OR (older patients) OR (65 years) OR (above 65 years) OR (> 65 years) OR (> 65) OR (≥ 65 years) OR (≥ 65) OR "aged"[Mesh] OR (aged, 80 and over[Mesh]) OR elderly OR frail OR (older adults))) AND  (("potentially inappropriate medication list"[Mesh]) OR (potentially inappropriate medication list) OR (BEERS list) OR (BEERS criteria) OR (STOPP) OR (STOPP list) OR (STOPP criteria) OR (priscus) OR (priscus list) OR (Priscuslist))) AND (((rehospitalization) OR (re-  hospitalization) OR (rehospitalisation) OR (re-hospitalisation) OR (re- admission) OR (readmission) OR (readmitted) OR (re-admitted) OR (emergency admission) OR (ed visits) OR (emergency visit) OR (drug- related hospital admissions) OR (DRA) OR (emergency revisit) OR (emergency revisits) OR (mortality[Mesh]) OR (death) OR (mortality) OR (mortal) OR (dead) OR (passed) OR (deceased) OR "patient readmission"[Mesh])) Sort by: Most Recent | Results  465 | Time  09:35:39 |
| --- | --- | --- | --- | --- | --- |
| #3 | … |  | Search: ((rehospitalization) OR (re-hospitalization) OR  (rehospitalisation) OR (re-hospitalisation) OR (re-admission) OR (readmission) OR (readmitted) OR (re-admitted) OR (emergency admission) OR (ed visits) OR (emergency visit) OR (drug-related hospital admissions) OR (DRA) OR (emergency revisit) OR  (emergency revisits) OR (mortality[Mesh]) OR (death) OR (mortality) OR (mortal) OR (dead) OR (passed) OR (deceased) OR "patient readmission"[Mesh]) Sort by: Most Recent | 2,771,987 | 09:34:52 |
| #2 | … |  | Search: ("potentially inappropriate medication list"[Mesh]) OR  (potentially inappropriate medication list) OR (BEERS list) OR (BEERS criteria) OR (STOPP) OR (STOPP list) OR (STOPP criteria) OR (priscus) OR (priscus list) OR (Priscuslist) Sort by: Most Recent | 2,779 | 09:34:28 |
| #1 | … |  | Search: ((geriatr*) OR (geriatry) OR (geriatric) OR (older patient) OR (older patients) OR (65 years) OR (above 65 years) OR (> 65 years) OR (> 65) OR (≥ 65 years) OR (≥ 65) OR "aged"[Mesh] OR (aged, 80 and over[Mesh]) OR elderly OR frail OR (older adults)) Sort by: Most Recent | 6,397,186 | 09:33:53 |

Showing 1 to 4 of 4 entries

### PubMed (German Search, 10.04.2024):


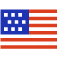
An official website of the United States government Here's how you know

**PubMed Advanced Search Builder**


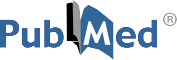


Add terms to the query box

All Fields

Enter a search term

ADD Show Index

Query box

Enter / edit your search query here

Search

**History and Search Details**

| Search  #5 | Actions  … | Details | Query  Search: ((((geriatr*) OR (geriatrie) OR (geriatrisch) OR (ältere Patienten) OR (älterer Patient) OR (65 Jahre) OR (über 65 Jahre) OR (> 65 Jahre) OR (> 65) OR (≥ 65) OR (alt) OR (betagt) OR (senior) OR (gebrechlich) OR (ältere Erwachsene) OR (alte Erwachsene))) AND (((potentiell ungeeignete medikation) OR (potentiell inadäquate  medikation) OR (potentiell unangebrachte medikation) OR (Priscusliste) OR (Priscus Liste) OR (Priscus Kriterium) OR (Priscus Kriterien)OR (BEERS Kriterium) OR (BEERS Kriterien) OR (BEERS Liste) OR (STOPP Kriterium) OR (STOPP Kriterien) OR (STOPP Liste)))) AND (((rehospitalisation) OR (re-hospitalisation) OR (rehospitalisierung)  OR (re-hospitalisierung) OR (Wiederaufnahme) OR  (Spitalwiederaufnahme) OR (krankenhauswiederaufnahme) OR (Krankenhauseinweisung) OR (spitaleinweisung) OR (ungeplanter Krankenheitsaufenthalt) OR (Notfallaufnahme) OR  (Notfallwiederaufnahme) OR (Besuch bei der Notfallaufnahme) OR  (notfallmässige Aufnahme) OR (notfallmässige Einweisung) OR (Tod)  OR (Versterben) OR (mortalität) OR (tödlich) OR (tot) OR (verstorben))) Sort by: Most Recent | Results  0 | Time  10:18:30 |
| --- | --- | --- | --- | --- | --- |
| #4 | … |  | Search: ((((geriatr*) OR (geriatrie) OR (geriatrisch) OR (ältere Patienten) OR (älterer Patient) OR (65 Jahre) OR (über 65 Jahre) OR (> 65 Jahre) OR (> 65) OR (≥ 65) OR (alt) OR (betagt) OR (senior) OR (gebrechlich) OR (ältere Erwachsene) OR (alte Erwachsene))) AND (((potentiell ungeeignete medikation) OR (potentiell inadäquate  medikation) OR (potentiell unangebrachte medikation) OR (Priscusliste) OR (Priscus Liste) OR (Priscus Kriterium) OR (Priscus Kriterien)OR (BEERS Kriterium) OR (BEERS Kriterien) OR (BEERS Liste) OR (STOPP Kriterium) OR (STOPP Kriterien) OR (STOPP Liste)))) AND (((rehospitalisation) OR (re-hospitalisation) OR (rehospitalisierung)  OR (re-hospitalisierung) OR (Wiederaufnahme) OR  (Spitalwiederaufnahme) OR (krankenhauswiederaufnahme) OR (Krankenhauseinweisung) OR (spitaleinweisung) OR (ungeplanter Krankenheitsaufenthalt) OR (Notfallaufnahme) OR | 0 | 10:18:30 |

### PubMed (German Search, 10.04.2024):

| Search | Actions | Details | Query  (Notfallwiederaufnahme) OR (Besuch bei der Notfallaufnahme) OR  (notfallmässige Aufnahme) OR (notfallmässige Einweisung) OR (Tod)  OR (Versterben) OR (mortalität) OR (tödlich) OR (tot) OR (verstorben))) - Schema: all Sort by: Most Recent | Results | Time |
| --- | --- | --- | --- | --- | --- |
| #3 | … |  | Search: ((rehospitalisation) OR (re-hospitalisation) OR (rehospitalisierung) OR (re-hospitalisierung) OR (Wiederaufnahme) OR (Spitalwiederaufnahme) OR (krankenhauswiederaufnahme) OR (Krankenhauseinweisung) OR (spitaleinweisung) OR (ungeplanter Krankenheitsaufenthalt) OR (Notfallaufnahme) OR  (Notfallwiederaufnahme) OR (Besuch bei der Notfallaufnahme) OR  (notfallmässige Aufnahme) OR (notfallmässige Einweisung) OR (Tod)  OR (Versterben) OR (mortalität) OR (tödlich) OR (tot) OR (verstorben)) Sort by: Most Recent | 11,038 | 10:14:03 |
| #2 | … |  | Search: ((potentiell ungeeignete medikation) OR (potentiell inadäquate medikation) OR (potentiell unangebrachte medikation) OR (Priscusliste) OR (Priscus Liste) OR (Priscus Kriterium) OR (Priscus Kriterien)OR (BEERS Kriterium) OR (BEERS Kriterien) OR (BEERS Liste) OR (STOPP Kriterium) OR (STOPP Kriterien) OR (STOPP Liste)) Sort by: Most Recent | 11 | 10:13:52 |
| #1 | … |  | Search: ((geriatr*) OR (geriatrie) OR (geriatrisch) OR (ältere Patienten) OR (älterer Patient) OR (65 Jahre) OR (über 65 Jahre) OR (> 65 Jahre) OR (> 65) OR (≥ 65) OR (alt) OR (betagt) OR (senior) OR (gebrechlich) OR (ältere Erwachsene) OR (alte Erwachsene)) Sort by: Most Recent | 429,386 | 10:13:31 |

Showing 1 to 5 of 5 entries

FOLLOW NCBI


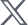

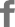

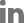

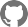

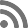


Connect with NLM


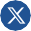


National Library of Medicine 8600 Rockville Pike

Bethesda, MD 20894

Web Policies FOIA

HHS Vulnerability Disclosure

Help Accessibility Careers

NLM NIH HHS USA.gov

### EMBASE:


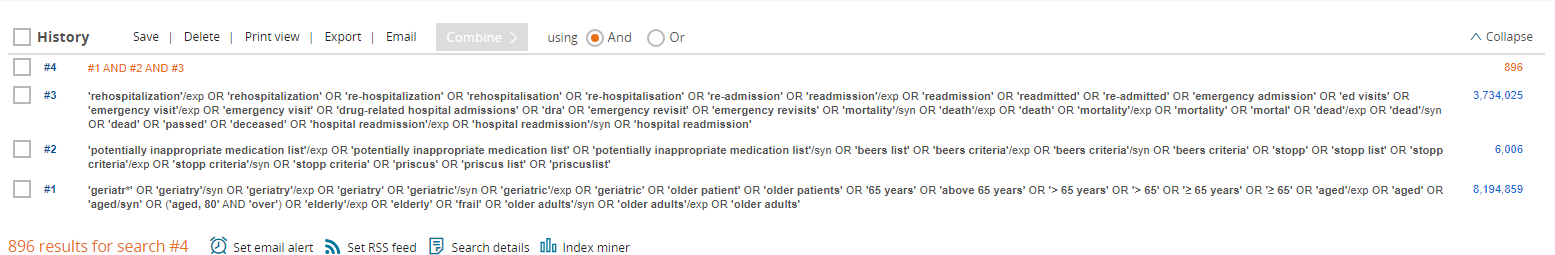


**EMBASE (German Search, 10.04.2024):**


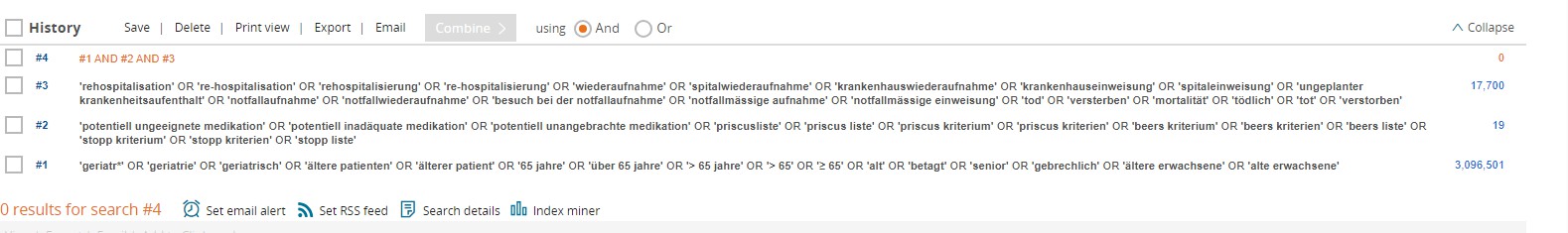


### CENTRAL:


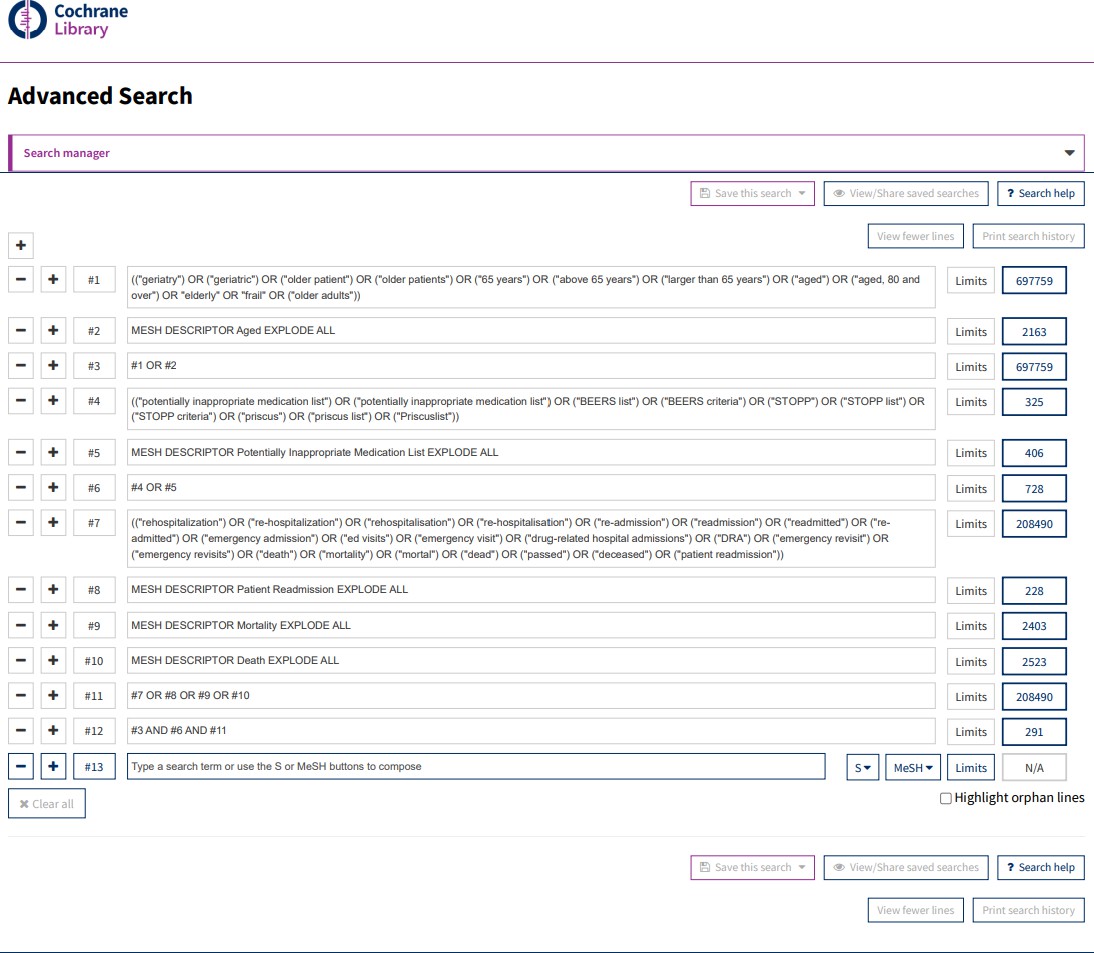


**CENTRAL (German Search, 10.04.2024):**


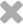


Clear all


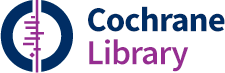


**Advanced Search**


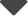


**Search manager**


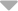


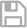
 Save this search


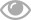


View/Share saved searches


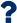


Search help


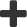


Print search history


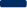

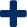


#1

(((geriatrie) OR (geriatrisch) OR (ältere Patienten) OR (älterer

Patient) OR (65 Jahre) OR (über 65 Jahre) OR (alt) OR (betagt) OR (senior) OR (gebrechlich) OR (ältere Erwachsene) OR (alte Erwachsene))):ti,ab,kw


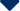


S

Limits

18519

(Word variations have been searched)


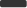

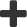


#2

((potentiell ungeeignete medikation) OR (potentiell inadäquate

medikation) OR (potentiell unangebrachte medikation) OR (Priscusliste) OR (Priscus Liste) OR (Priscus Kriterium) OR (Priscus Kriterien) OR (BEERS Kriterium) OR (BEERS Kriterien) OR (BEERS Liste) OR

(STOPP Kriterium) OR (STOPP Kriterien) OR (STOPP Liste))

Limits

0


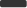

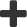


#3

((rehospitalisation) OR (re-hospitalisation) OR (rehospitalisierung) OR

(re-hospitalisierung) OR (Wiederaufnahme) OR (Spitalwiederaufnahme)

OR (krankenhauswiederaufnahme) OR (Krankenhauseinweisung) OR (spitaleinweisung) OR (ungeplanter Krankenheitsaufenthalt) OR

(Notfallaufnahme) OR (Notfallwiederaufnahme) OR (Besuch bei der Notfallaufnahme) OR (notfallmässige Aufnahme) OR (notfallmässige

Einweisung) OR (Tod) OR (Versterben) OR (mortalität) OR (tödlich) OR (tot) OR (verstorben))

Limits

2066


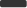

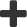


#4

#1 AND #2 AND #3

Limits

0


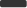

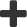


#5


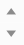


Type a search term or use the S or MeSH


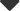


S


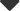


MeSH

Limits

N/A


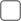
 Highlight orphan lines


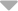


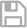
 Save this search


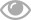


View/Share saved searches


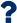


Search help

Print search history

## Update Search 17.05.2024

###
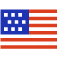
PubMed (with filter publication date 05.04-18.05.2024):

An official website of the United States government Here's how you know

**PubMed Advanced Search Builder**


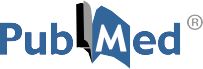


Filters applied: From 2024/4/5 to 2024/5/18. Clear all

Add terms to the query box

All Fields

Enter a search term

ADD Show Index

Query box

Enter / edit your search query here

Search

**History and Search Details**

| Search  #5 | Actions  … | Details | Query  Search: ((((geriatr*) OR (geriatry) OR (geriatric) OR (older patient) OR (older patients) OR (65 years) OR (above 65 years) OR (> 65 years) OR (> 65) OR (≥ 65 years) OR (≥ 65) OR "aged"[Mesh] OR (aged, 80 and over[Mesh]) OR elderly OR frail OR (older adults))) AND  (("potentially inappropriate medication list"[Mesh]) OR (potentially inappropriate medication list) OR (BEERS list) OR (BEERS criteria) OR (STOPP) OR (STOPP list) OR (STOPP criteria) OR (priscus) OR (priscus list) OR (Priscuslist))) AND (((rehospitalization) OR (re-  hospitalization) OR (rehospitalisation) OR (re-hospitalisation) OR (re- admission) OR (readmission) OR (readmitted) OR (re-admitted) OR (emergency admission) OR (ed visits) OR (emergency visit) OR (drug- related hospital admissions) OR (DRA) OR (emergency revisit) OR (emergency revisits) OR (mortality[Mesh]) OR (death) OR (mortality) OR (mortal) OR (dead) OR (passed) OR (deceased) OR "patient readmission"[Mesh])) Filters: from 2024/4/6 - 2024/5/17 Sort by: Most Recent | Results  4 | Time  05:18:33 |
| --- | --- | --- | --- | --- | --- |
| #4 | … |  | Search: ((((geriatr*) OR (geriatry) OR (geriatric) OR (older patient) OR (older patients) OR (65 years) OR (above 65 years) OR (> 65 years) OR (> 65) OR (≥ 65 years) OR (≥ 65) OR "aged"[Mesh] OR (aged, 80 and over[Mesh]) OR elderly OR frail OR (older adults))) AND  (("potentially inappropriate medication list"[Mesh]) OR (potentially inappropriate medication list) OR (BEERS list) OR (BEERS criteria) OR (STOPP) OR (STOPP list) OR (STOPP criteria) OR (priscus) OR (priscus list) OR (Priscuslist))) AND (((rehospitalization) OR (re-  hospitalization) OR (rehospitalisation) OR (re-hospitalisation) OR (re- admission) OR (readmission) OR (readmitted) OR (re-admitted) OR (emergency admission) OR (ed visits) OR (emergency visit) OR (drug- related hospital admissions) OR (DRA) OR (emergency revisit) OR (emergency revisits) OR (mortality[Mesh]) OR (death) OR (mortality) | 472 | 05:17:07 |

### PubMed (with filter publication date 05.04-18.05.2024):

| Search | Actions | Details | Query  OR (mortal) OR (dead) OR (passed) OR (deceased) OR "patient readmission"[Mesh])) Sort by: Most Recent | Results | Time |
| --- | --- | --- | --- | --- | --- |
| #3 | … |  | Search: ((rehospitalization) OR (re-hospitalization) OR  (rehospitalisation) OR (re-hospitalisation) OR (re-admission) OR (readmission) OR (readmitted) OR (re-admitted) OR (emergency admission) OR (ed visits) OR (emergency visit) OR (drug-related hospital admissions) OR (DRA) OR (emergency revisit) OR  (emergency revisits) OR (mortality[Mesh]) OR (death) OR (mortality) OR (mortal) OR (dead) OR (passed) OR (deceased) OR "patient readmission"[Mesh]) Sort by: Most Recent | 2,789,957 | 05:16:35 |
| #2 | … |  | Search: ("potentially inappropriate medication list"[Mesh]) OR  (potentially inappropriate medication list) OR (BEERS list) OR (BEERS criteria) OR (STOPP) OR (STOPP list) OR (STOPP criteria) OR (priscus) OR (priscus list) OR (Priscuslist) Sort by: Most Recent | 2,806 | 05:16:17 |
| #1 | … |  | Search: ((geriatr*) OR (geriatry) OR (geriatric) OR (older patient) OR (older patients) OR (65 years) OR (above 65 years) OR (> 65 years) OR (> 65) OR (≥ 65 years) OR (≥ 65) OR "aged"[Mesh] OR (aged, 80 and over[Mesh]) OR elderly OR frail OR (older adults)) Sort by: Most Recent | 6,427,299 | 05:15:41 |

Showing 1 to 5 of 5 entries

FOLLOW NCBI


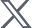

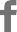

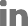

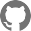

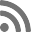


Connect with NLM


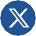


National Library of Medicine 8600 Rockville Pike

Bethesda, MD 20894

Web Policies FOIA

HHS Vulnerability Disclosure

Help Accessibility Careers

NLM NIH HHS USA.gov

### PubMed (German Search, 17.05.2024):


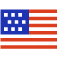
An official website of the United States government Here's how you know

**PubMed Advanced Search Builder**


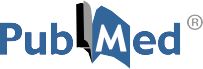


Add terms to the query box

All Fields

Enter a search term

ADD Show Index

Query box

Enter / edit your search query here

Search

**History and Search Details**

| Search  #5 | Actions  … | Details | Query  Search: ((((geriatr*) OR (geriatrie) OR (geriatrisch) OR (ältere Patienten) OR (älterer Patient) OR (65 Jahre) OR (über 65 Jahre) OR (> 65 Jahre) OR (> 65) OR (≥ 65) OR (alt) OR (betagt) OR (senior) OR (gebrechlich) OR (ältere Erwachsene) OR (alte Erwachsene))) AND (((potentiell ungeeignete medikation) OR (potentiell inadäquate  medikation) OR (potentiell unangebrachte medikation) OR (Priscusliste) OR (Priscus Liste) OR (Priscus Kriterium) OR (Priscus Kriterien)OR (BEERS Kriterium) OR (BEERS Kriterien) OR (BEERS Liste) OR (STOPP Kriterium) OR (STOPP Kriterien) OR (STOPP Liste)))) AND (((rehospitalisation) OR (re-hospitalisation) OR (rehospitalisierung)  OR (re-hospitalisierung) OR (Wiederaufnahme) OR  (Spitalwiederaufnahme) OR (krankenhauswiederaufnahme) OR (Krankenhauseinweisung) OR (spitaleinweisung) OR (ungeplanter Krankenheitsaufenthalt) OR (Notfallaufnahme) OR  (Notfallwiederaufnahme) OR (Besuch bei der Notfallaufnahme) OR  (notfallmässige Aufnahme) OR (notfallmässige Einweisung) OR (Tod)  OR (Versterben) OR (mortalität) OR (tödlich) OR (tot) OR (verstorben))) Sort by: Most Recent | Results  0 | Time  05:32:13 |
| --- | --- | --- | --- | --- | --- |
| #4 | … |  | Search: ((((geriatr*) OR (geriatrie) OR (geriatrisch) OR (ältere Patienten) OR (älterer Patient) OR (65 Jahre) OR (über 65 Jahre) OR (> 65 Jahre) OR (> 65) OR (≥ 65) OR (alt) OR (betagt) OR (senior) OR (gebrechlich) OR (ältere Erwachsene) OR (alte Erwachsene))) AND (((potentiell ungeeignete medikation) OR (potentiell inadäquate  medikation) OR (potentiell unangebrachte medikation) OR (Priscusliste) OR (Priscus Liste) OR (Priscus Kriterium) OR (Priscus Kriterien)OR (BEERS Kriterium) OR (BEERS Kriterien) OR (BEERS Liste) OR (STOPP Kriterium) OR (STOPP Kriterien) OR (STOPP Liste)))) AND (((rehospitalisation) OR (re-hospitalisation) OR (rehospitalisierung)  OR (re-hospitalisierung) OR (Wiederaufnahme) OR  (Spitalwiederaufnahme) OR (krankenhauswiederaufnahme) OR (Krankenhauseinweisung) OR (spitaleinweisung) OR (ungeplanter Krankenheitsaufenthalt) OR (Notfallaufnahme) OR | 0 | 05:32:13 |

### PubMed (German Search, 17.05.2024):

| Search | Actions | Details | Query  (Notfallwiederaufnahme) OR (Besuch bei der Notfallaufnahme) OR  (notfallmässige Aufnahme) OR (notfallmässige Einweisung) OR (Tod)  OR (Versterben) OR (mortalität) OR (tödlich) OR (tot) OR (verstorben))) - Schema: all Sort by: Most Recent | Results | Time |
| --- | --- | --- | --- | --- | --- |
| #3 | … |  | Search: ((rehospitalisation) OR (re-hospitalisation) OR (rehospitalisierung) OR (re-hospitalisierung) OR (Wiederaufnahme) OR (Spitalwiederaufnahme) OR (krankenhauswiederaufnahme) OR (Krankenhauseinweisung) OR (spitaleinweisung) OR (ungeplanter Krankenheitsaufenthalt) OR (Notfallaufnahme) OR  (Notfallwiederaufnahme) OR (Besuch bei der Notfallaufnahme) OR  (notfallmässige Aufnahme) OR (notfallmässige Einweisung) OR (Tod)  OR (Versterben) OR (mortalität) OR (tödlich) OR (tot) OR (verstorben)) Sort by: Most Recent | 11,099 | 05:31:51 |
| #2 | … |  | Search: ((potentiell ungeeignete medikation) OR (potentiell inadäquate medikation) OR (potentiell unangebrachte medikation) OR (Priscusliste) OR (Priscus Liste) OR (Priscus Kriterium) OR (Priscus Kriterien)OR (BEERS Kriterium) OR (BEERS Kriterien) OR (BEERS Liste) OR (STOPP Kriterium) OR (STOPP Kriterien) OR (STOPP Liste)) Sort by: Most Recent | 11 | 05:31:28 |
| #1 | … |  | Search: ((geriatr*) OR (geriatrie) OR (geriatrisch) OR (ältere Patienten) OR (älterer Patient) OR (65 Jahre) OR (über 65 Jahre) OR (> 65 Jahre) OR (> 65) OR (≥ 65) OR (alt) OR (betagt) OR (senior) OR (gebrechlich) OR (ältere Erwachsene) OR (alte Erwachsene)) Sort by: Most Recent | 432,739 | 05:31:05 |

Showing 1 to 5 of 5 entries

FOLLOW NCBI


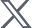

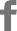

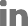

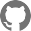

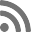


Connect with NLM


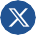


National Library of Medicine 8600 Rockville Pike

Bethesda, MD 20894

Web Policies FOIA

HHS Vulnerability Disclosure

Help Accessibility Careers

NLM NIH HHS USA.gov

**EMBASE (with filter publication date 06.04.-17.05.2023):**


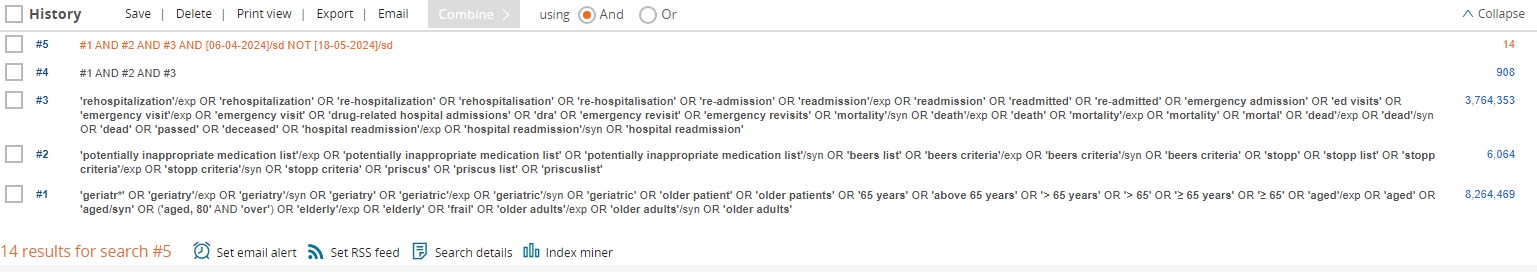


**EMBASE (German Search, 17.05.2024):**


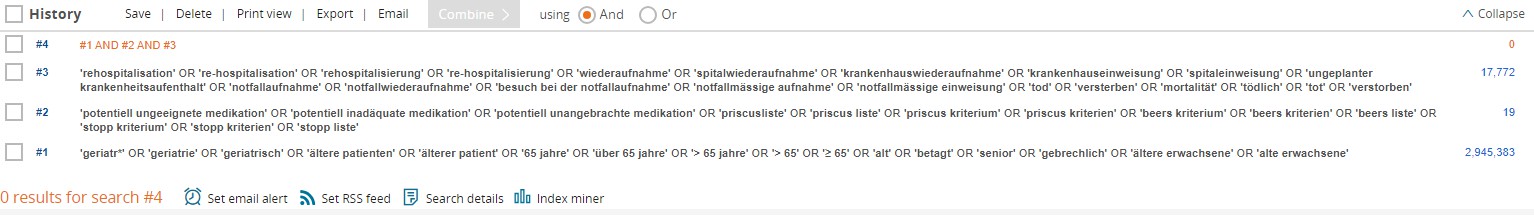


**CENTRAL:**


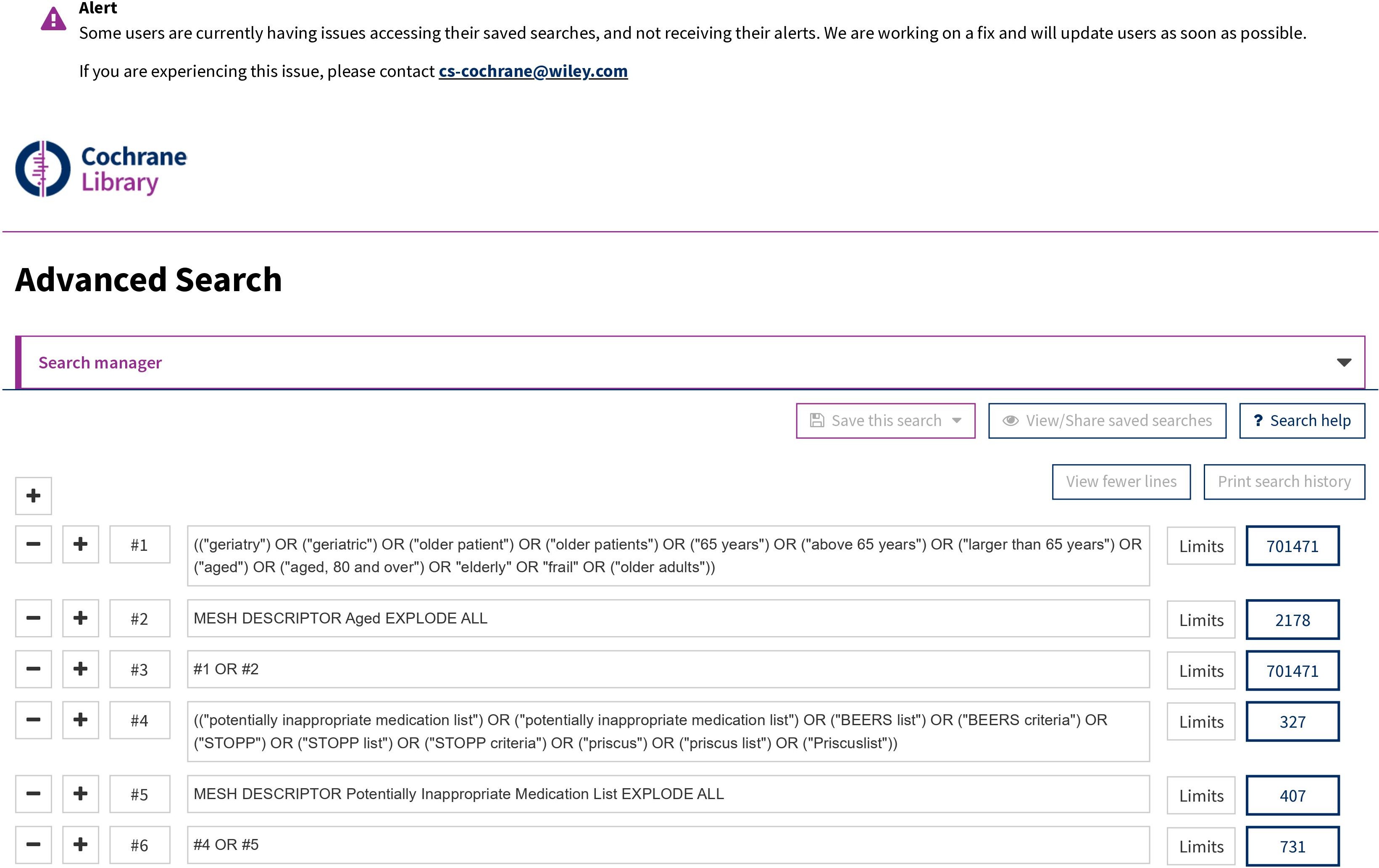


**CENTRAL:**


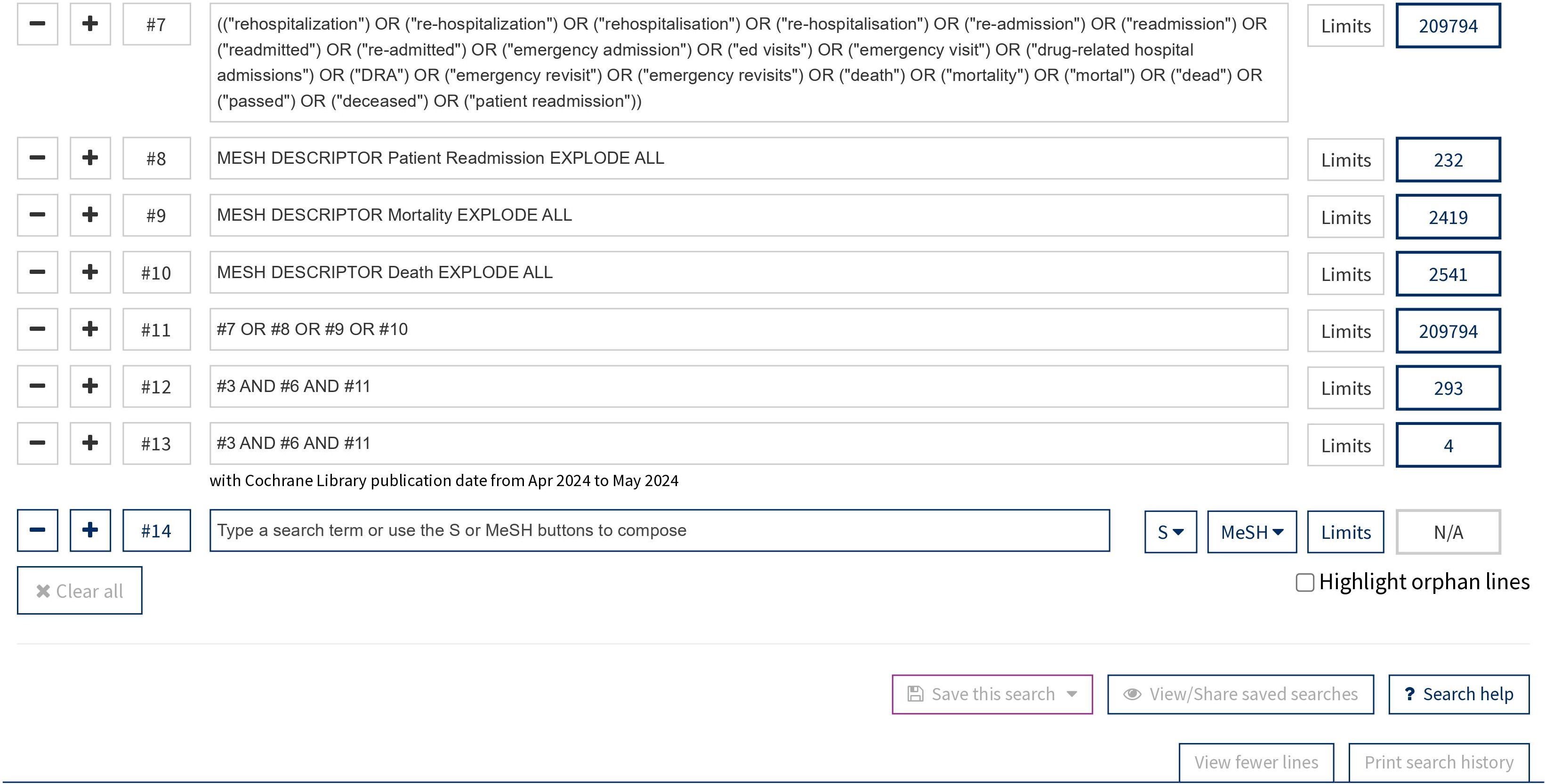


**CENTRAL (German Search, 17.05.2024):**


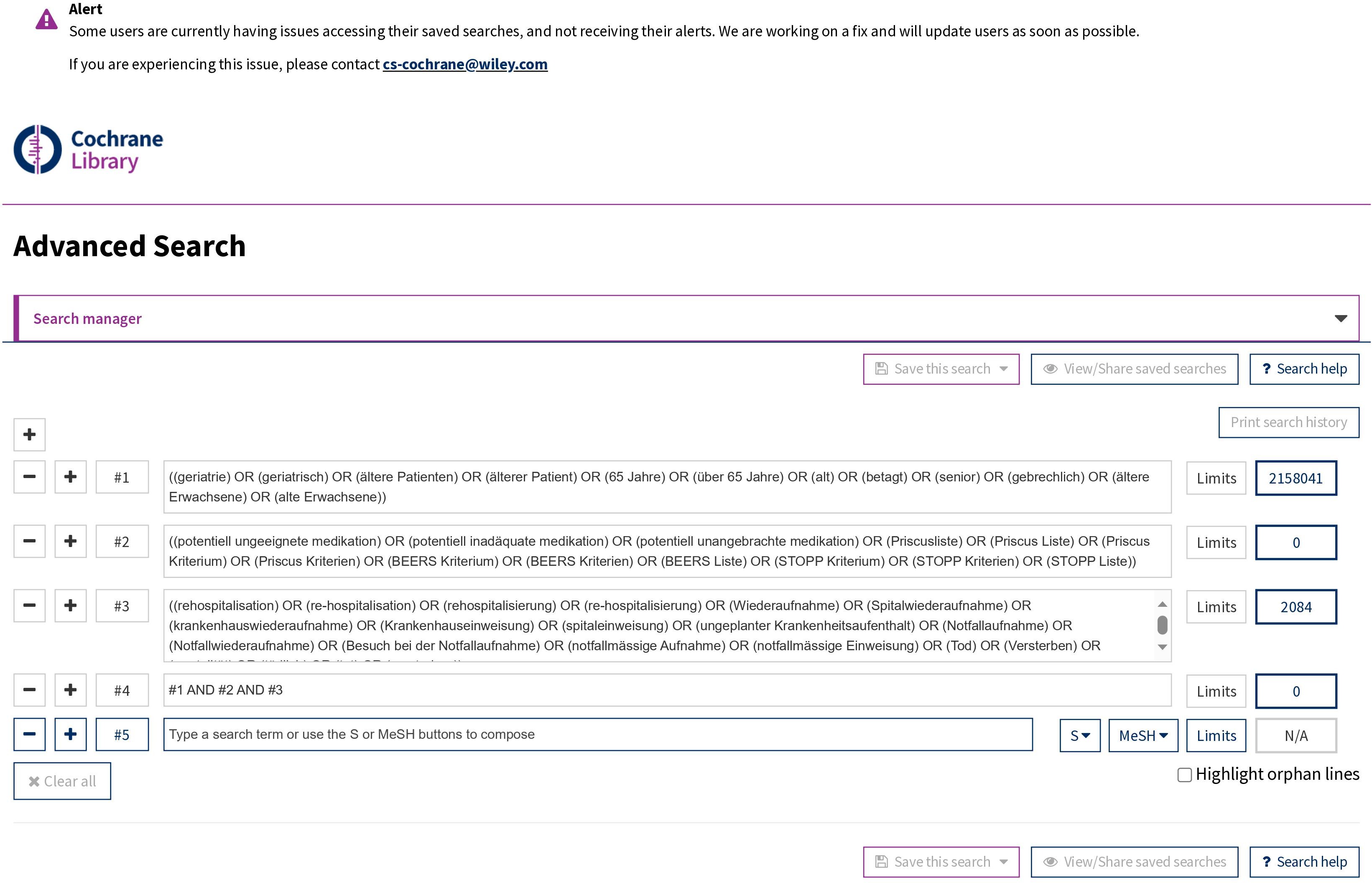


## S3- Reasons for Exclusion of Articles in Full Text Review

**Author, Year Title Reasons Exclusion**

Santolaya-Perr´ın 2016

A randomised controlled trial on the efficacy of a multi- disciplinary health care team on morbidity and mortality of el- derly patients attending the Emergency Department. Study design and preliminary results

Only PIM prevalence, no systematic analysis for detection of PIMs (i.e., PIPs), outcome at 12 months.

Trenaman 2017 Antipsychotic drug use by community dwelling older persons and adherence to stopp criteria after a fall

Chin 1999 Appropriateness of medication selection for older persons in an urban academic emergency department

Publication not found, presumably conference poster.

Outcome missing.

VanderLinden 2017

Combined Use of the Rationalization of Home Medication by an Adjusted STOPP in Older Patients (RASP) List and a Pharmacist-Led Medication Review in Very Old Inpatients: impact on Quality of Prescribing and Clinical Outcome

Composite outcome of drug discontinuation and dose reduction.

Spinewine 2007 Effect of a collaborative approach on the quality of prescrib- ing for geriatric inpatients: a randomised, controlled trial

Kimura 2022 Effectiveness of pharmacist intervention for deprescribing potentially inappropriate medications: a prospective observational study

Cohen 2022 Evaluating Potentially Inappropriate Medications in Older Kidney Transplant Recipients

Lai 2016 Impact of potentially inappropriate medication and continuity of care in a sample of Taiwan elderly patients with diabetes mellitus who have also experienced heart failure

Mansur 2009 Is there an association between inappropriate prescription drug use and adherence in discharged elderly patients?

Blackwell 2009 National study of medications associated with injury in elderly Medicare/Medicaid dual enrollees during 2003

Forget 2020 Potentially Inappropriate Medication Use in Older Adults in the Preoperative Period: A Retrospective Study of a Non- cardiac Surgery Cohort

Buda 2020 Potentially inappropriate prescriptions in ambulatory elderly patients living in rural areas of Romania using stopp/start (Version 2) criteria

No outcome on hospitalisation or mortality reported.

Pharmacist intervention and active change of medication

Missing data: total number of readmissions per group not reported.

Missing data: no data on readmission or mor- tality at 3 months.

Data 1 month after discharge.

Outcomes per prescription (re-)fills, not pa- tients.

Missing data, outcome is postoperative length or stay.

Only prevalence of PIMs, no outcome at 3 months.

VanDerLinden 2014

Reduction of inappropriate prescribing in older persons using the RASP list: a cluster-randomised controlled trial

Conference poster.

Ishii 2017 The association of change in medication regimen and use of inappropriate medication based on beers criteria with adverse outcomes in Japanese long-term care facilities

Hyttinen 2019 The association of potentially inappropriate medication use on health outcomes and hospital costs in community- dwelling older persons: a longitudinal 12-year study

Boersma 2019 The effect of providing prescribing recommendations on appropriate prescribing: A cluster-randomised controlled trial in older adults in a preoperative setting

Outcome is admission to geriatric facility to hospita-lisation, not hospitalisation to hospital- isation. No stratification on admission from hospital for outcome.

Outcome is number of fractures.

Outcome is number of medication changes.

Santolaya-Perr´ın 2019

Pardo-Cabello 2022

The efficacy of a medication review programme conducted in an emergency department

The impact of PIPs on mortality and readmissions in older adults: a retrospective cohort study

Outcome is number of medication changes. Readmission/mortality rates up to 2 years.

Viana 2022 Use of potentially inappropriate medications and adverse events in older outpatients with acute conditions

Hsieh 2023 Using PIM-Taiwan, PRISCUS, and Beers criteria to assess potentially inappropriate medication use among older adults with 90-day rehospitalisation: a population-based study in Taiwan

Population *≥*60 years.

Readmission rates only given stratified by PIM group by organ system, not overall.

Abbrevations: AE = Adverse Events, ADE = Adverse Drug Event, ED = Emergency Department, ICU = Intensive Care Unit, PIM

= Potentially Inappropriate Medication, PIP = Potentially Inappropriate Prescription

## S4- ROBINS-I Judgement

### Brown 2016

Table S4: Risk of Bias assessment of study Brown 2016 by application of ROBINS-I tool

| **Domain** | **Outcome 1:**  **Rehospitalisation within 30 days** |
| --- | --- |
| Bias due to confounding | Moderate risk |
| Bias in selection of participants  into the study | Low risk |
| Bias in classification of  interventions | Low risk |
| Bias due to deviations from  intended interventions | Low risk |
| Bias due to missing data | Low risk |
| Bias in measurement of  outcomes | Low risk |
| Bias in selection of the reported  results | Low risk |
| **Overall** | Moderate risk |

### D’Aiuto 2024

Table S5: Risk of Bias assessment of study D’Aiuto 2024 by application of ROBINS-I tool

| **Domain** | **Outcome 2:**  **Mortality within 30 days** |
| --- | --- |
| Bias due to confounding | Moderate risk |
| Bias in selection of participants  into the study | Low risk |
| Bias in classification of  interventions | Low risk |
| Bias due to deviations from  intended interventions | Low risk |
| Bias due to missing data | Low risk |
| Bias in measurement of  outcomes | Low risk |
| Bias in selection of the reported  results | Low risk |
| **Overall** | Moderate risk |

### DeVincentis 2022

Table S6: Risk of Bias assessment of study DeVincentis 2022 by application of ROBINS-I tool

| **Domain** | **Outcome 1:**  **Rehospitalisation within 30 days** | **Outcome 2:**  **Mortality within 30 days** |
| --- | --- | --- |
| Bias due to confounding | Moderate risk | Moderate risk |
| Bias in selection of participants  into the study | Low risk | Low risk |
| Bias in classification of  interventions | Low risk | Low risk |
| Bias due to deviations from  intended interventions | Low risk | Low risk |
| Bias due to missing data | Low risk | Low risk |
| Bias in measurement of  outcomes | Low risk | Low risk |
| Bias in selection of the reported  results | Low risk | Low risk |
| **Overall** | Moderate risk | Moderate risk |

### Fabbietti 2018

Table S7: Risk of Bias assessment of study Fabbietti 2018 by application of ROBINS-I tool

| **Domain** | **Outcome 1:**  **Rehospitalisation within 30 days** |
| --- | --- |
| Bias due to confounding | Serious risk |
| Bias in selection of participants  into the study | Low risk |
| Bias in classification of  interventions | Low risk |
| Bias due to deviations from  intended interventions | Low risk |
| Bias due to missing data | Serious risk |
| Bias in measurement of  outcomes | Low risk |
| Bias in selection of the reported  results | Low risk |
| **Overall** | Serious risk |

### Liang 2023

Table S8: Risk of Bias assessment of study Liang 2023 by application of ROBINS-I tool

| **Domain** | **Outcome 1:**  **Rehospitalisation within 30 days** |
| --- | --- |
| Bias due to confounding | Moderate risk |
| Bias in selection of participants  into the study | Low risk |
| Bias in classification of  interventions | Low risk |
| Bias due to deviations from  intended interventions | Low risk |
| Bias due to missing data | Low risk |
| Bias in measurement of  outcomes | Low risk |
| Bias in selection of the reported  results | Moderate risk |
| **Overall** | Moderate risk |

### Mekonnen 2022

Table S9: Risk of Bias assessment of study Mekonnen 2022 by application of ROBINS-I tool

| **Domain** | **Outcome 1:**  **Rehospitalisation within 30 days** | **Outcome 2:**  **Mortality within 30 days** |
| --- | --- | --- |
| Bias due to confounding | Serious risk | Serious risk |
| Bias in selection of participants  into the study | Low risk | Low risk |
| Bias in classification of  interventions | Low risk | Low risk |
| Bias due to deviations from  intended interventions | Low risk | Low risk |
| Bias due to missing data | Low risk | Low risk |
| Bias in measurement of  outcomes | Moderate risk | Moderate risk |
| Bias in selection of the reported  results | Low risk | Low risk |
| **Overall** | Serious risk | Serious risk |

### Mekonnen 2023

Table S10: Risk of Bias assessment of study Mekonnen 2023 by application of ROBINS-I tool

| **Domain** | **Outcome 1:**  **Rehospitalisation within 30 days** | **Outcome 2:**  **Mortality within 30 days** |
| --- | --- | --- |
| Bias due to confounding | Serious risk | Serious risk |
| Bias in selection of participants  into the study | Low risk | Low risk |
| Bias in classification of  interventions | Low risk | Low risk |
| Bias due to deviations from  intended interventions | Low risk | Low risk |
| Bias due to missing data | Serious risk | Serious risk |
| Bias in measurement of  outcomes | Serious risk | Serious risk |
| Bias in selection of the reported  results | Moderate risk | Moderate risk |
| **Overall** | Serious risk | Serious risk |

### Pasina 2014

Table S11: Risk of Bias assessment of study Pasina 2014 by application of ROBINS-I tool

| **Domain** | **Outcome 1:**  **Rehospitalisation within 30 days** | **Outcome 2:**  **Mortality within 30 days** |
| --- | --- | --- |
| Bias due to confounding | Serious risk | Serious risk |
| Bias in selection of participants  into the study | Low risk | Low risk |
| Bias in classification of  interventions | Low risk | Low risk |
| Bias due to deviations from  intended interventions | Low risk | Low risk |
| Bias due to missing data | Serious risk | Serious risk |
| Bias in measurement of  outcomes | Low risk | Low risk |
| Bias in selection of the reported  results | Serious risk | Serious risk |
| **Overall** | Serious risk | Serious risk |

### Renom-Guiteras 2018

Table S12: Risk of Bias assessment of study Renom-Guiteras 2018 by application of ROBINS-I tool

| **Domain** | **Outcome 1:**  **Rehospitalisation within 30 days** | **Outcome 2:**  **Mortality within 30 days** |
| --- | --- | --- |
| Bias due to confounding | Serious risk | Serious risk |
| Bias in selection of participants  into the study | Low risk | Low risk |
| Bias in classification of  interventions | Low risk | Low risk |
| Bias due to deviations from  intended interventions | Low risk | Low risk |
| Bias due to missing data | Moderate risk | Moderate risk |
| Bias in measurement of  outcomes | Low risk | Low risk |
| Bias in selection of the reported  results | Serious risk | Serious risk |
| **Overall** | Serious risk | Serious risk |

## S5- Summary of findings

Outcome Rehospitalisation within three months:

### Study Population Intervention Outcome

| **Study** | **Country/ Study period** | **Study Design** | **Sample size (% female)** | **Mean age** *±* **SD** | **Population inclusion** | **Screening Tool (version)** | **Outcome description** | **OR / 95% CI** | **Significance: (OR**  **= 1 not in 90% CI / P-value** |
| --- | --- | --- | --- | --- | --- | --- | --- | --- | --- |
| Mekonnen 2022 | Australia/ | Retrospective cohort | 232 | 80.0 *±* | *≥*65 years, with hospital stay *≥*1 day, | STOPP (v2) | Hospital readmissions, ED presen- | 4.87 / 2.52-9.40 | yes / n.a. |
|  | 03.2021- | study (followed by | (51.7%) | 7.9 | consecutively discharged alive to usual |  | tations, and composite readmissions |  |  |
|  | 08.2021 | phone interviews) |  |  | residency |  | and/or ED presentations within 3 |  |  |
|  |  |  |  |  |  |  | months of index hospitalisation |  |  |
| Mekonnen 2023 | Australia/ | Prospective observa- | 1890 | 82.6 *±* | From RESORT cohort; Admitted to | STOPP (v2) | Hospital readmissions (i.e., unplanned | 1.37 / 1.09-1.71 | yes / 0.006 |
|  | 10.2017- | tional study (followed | (56.3%) | 8.1 | geriatric rehabilitation, informed con- |  | acute readmission after discharge) |  |  |
|  | 03.2020 | by phone interviews) |  |  | sent |  | and mortality at 3 or 12 months post- |  |  |
|  |  |  |  |  |  |  | discharge |  |  |
| Fabbietti 2018 | Italy/ | Prospective observa- | 647 | 80.1 *±* | *≥*65 years admitted to any of the wards | Beers (2015), | Any admission during 3-month follow | Beers: 1.09 / 0.66-1.82 | Beers: no / n.a. |
|  | 01.2023- | tional study | (49.0%) | 6.9 |  | STOPP (v2) | up after discharge | STOPP: 1.25 / 0.76-2.04 | STOPP: no / n.a. |
|  | 12.2013 |  |  |  |  |  |  |  |  |
| Renom- | 8 European | Prospective survey | 2004 | 83.0 *±* | *≥*65 years with diagnosis of demetia, | EU(7)-PIM list | Mortality, fall-related injury or hos- | 1.00 / 0.70-1.41 | no / n.a. |
| Guiteras 2018 | countries*/ |  | (67.5%) | 6.6 | 24 or lower points in standardised mini |  | pitalisation after 3 months |  |  |
|  | 11.2010- |  |  |  | mental state examination, with an infor- |  |  |  |  |
|  | 04.2012 |  |  |  | mal caregiver |  |  |  |  |
| DeVincentis | Italy/ | Prospective | 2631 | median | *≥*65 years who were discharged at | Beers (2019), | Survival, occurrence of | Beers: 0.99 / 0.79-1.24 | Beers: no / n.a. |
| 2020 | 01.2010- | cohort study | (51.4%) | (IQR): | home, from REPOSI cohort | STOPP (v2) | rehospitalisation and functional status | STOPP: 1.05 / 0.83-1.33 | STOPP: no / n.a. |
|  | 12.2016 |  |  | 79.6 *±* |  |  | decline at 3 months from discharge |  |  |
|  |  |  |  | 12 |  |  |  |  |  |
| Brown 2016 | USA/ | Retrospective | 174275 |  | *≥*65 years, at least 9 months of conti- | Beers (2003), | ADEs, all-cause ED visits, or all-cause | Beers 03: 2.31 / 2.26-2.37 | Beers 03: yes / n.a. |
|  | 01.2006- | cohort study | (54.3%) |  | nuous medical and pharmacy cove- | Beers (2012), | hospitalisations in following month | Beers 12: 2.25 / 2.20-2.30 | Beers 12: yes / n.a. |
|  | 12.2009 |  |  |  | rage including 6 months pre-index | STOPP (v1) | after PIM exposure | STOPP: 2.80 / 2.74-2.87 | STOPP: yes / n.a. |
|  |  |  |  |  | period and 3 months follow-up |  |  |  |  |
| Pasina 2014 | Italy/ | Prospective cross- | 844 | 78.8 *±* | *≥*65 years, signed informed consent, | Beers (2003), | Prevalence of PIMs, risk of AEs | Beers 03: 0.87 / 0.54-1.38 | Beers 03: no / n.a. |
|  | 01.2010- | sectional | (51.2%) | 7.4 | first 10 patients of each month from | Beers (2012) | from discharge to follow-up date (3 | Beers 12: 0.85 / 0.55-1.32 | Beers 12: no / n.a. |
|  | 12.2010 |  |  |  | REPOSI cohort |  | months), rehospitalisation and all- |  |  |
|  |  |  |  |  |  |  | cause mortality at follow-up (3 months) |  |  |
| Liang 2023 | Taiwan, | Retrospective cohort | 2671 | 77.2 *±* | *≥*65 years admitted to internal | Beers (2015) | ED room revisits and readmissions at | 1.27 / 1.07-1.50 | yes / n.a. |
|  | 04.2017- | study | (39.6%) | 8.4 | medicine ward for the first time in |  | 1, 3, and 6 months postdischarge |  |  |
|  | 12.2017 |  |  |  | study period, no hospice or limited life |  |  |  |  |
|  |  |  |  |  | expectancy due to terminal illness, not |  |  |  |  |
|  |  |  |  |  | in ICU |  |  |  |  |

Abbrevations: AE = Adverse Events, ADE = Adverse Drug Event, ED = Emergency Department, ICU = Intensive Care Unit, IQR = Interquartile Range, SD = Standard Deviation.

*England, Estonia, Finland, France, Germany, the Netherlands, Spain and Sweden

Outcome Death within three months:

### Study Population Intervention Outcome

| **Study** | **Country/ Study period** | **Study Design** | **Sample size (% female)** | **Mean age** *±* **SD** | **Population inclusion** | **Screening Tool (version)** | **Outcome description** | **OR / 95% CI** | **Significance: (OR**  **= 1 not in 90% CI / P-value** |
| --- | --- | --- | --- | --- | --- | --- | --- | --- | --- |
| Mekonnen 2022 | Australia/ | Retrospective cohort | 232 | 80.0 *±* | *≥*65 years, with hospital stay *≥*1 day, | STOPP (v2) | Hospital readmissions, ED presen- | 0.94 / 0.35-2.52 | no / n.a. |
|  | 03.2021- | study (followed by | (51.7%) | 7.9 | consecutively discharged alive to usual |  | tations, and composite readmissions |  |  |
|  | 08.2021 | phone interviews) |  |  | residency |  | and/or ED presentations within 3 |  |  |
|  |  |  |  |  |  |  | months of index hospitalisation |  |  |
| Mekonnen 2023 | Australia/ | Prospective | 1890 | 82.6 *±* | From RESORT cohort; Admitted to | STOPP (v2) | Hospital readmissions (i.e., unplanned | 1.09 / 0.76-1.55 | no / 0.636 |
|  | 10.2017- | observational study | (56.3%) | 8.1 | geriatric rehabilitation, informed con- |  | acute readmission after discharge) |  |  |
|  | 03.2020 | (followed by phone |  |  | sent |  | and mortality at 3 or 12 months post- |  |  |
|  |  | interviews) |  |  |  |  | discharge |  |  |
| Renom- | 8 European | Prospective survey | 2004 | 83.0 *±* | *≥*65 years with diagnosis of demetia, | EU(7)-PIM list | Mortality, fall-related injury or hos- | 0.86 / 0.53-1.40 | no / n.a. |
| Guiteras 2018 | countries*/ |  | (67.5%) | 6.6 | 24 or lower points in standardised mini |  | pitalisation after 3 months |  |  |
|  | 11.2010- |  |  |  | mental state examination, with an infor- |  |  |  |  |
|  | 04.2012 |  |  |  | mal caregiver |  |  |  |  |
| DeVincentis | Italy/ | Prospective cohort | 2631 | median | *≥*65 years who were discharged at | Beers (2019), | Survival, occurrence of rehospitali- | Beers: 1.00 / 0.73-1.37 | Beers: no / n.a. |
| 2020 | 01.2010- | study | (51.4%) | (IQR): | home, from REPOSI cohort | STOPP (v2) | sation and functional status decline at | STOPP: 1.20 / 0.87-1.66 | STOPP: no / n.a. |
|  | 12.2016 |  |  | 79.6 *±* |  |  | 3 months from discharge |  |  |
|  |  |  |  | 12 |  |  |  |  |  |
| Pasina 2014 | Italy/ | Prospective cross- | 844 | 78.8 *±* | *≥*65 years, signed informed consent, | Beers (2003), | Prevalence of PIMs, risk of AEs | Beers 03: 0.90 / 0.46-1.78 | Beers 03: no / n.a. |
|  | 01.2010- | sectional | (51.2%) | 7.4 | first 10 patients of each month from | Beers (2012) | from discharge to follow-up date (3 | Beers 12: 1.02 / 0.56-1.87 | Beers 12: no / n.a. |
|  | 12.2010 |  |  |  | REPOSI cohort |  | months), rehospitalisation and all- |  |  |
|  |  |  |  |  |  |  | cause mortality at follow-up (3 months) |  |  |
| D’Aiuto 2024 | Canada/ | Retrospective nested | 200 | 76.7 | *≥*65 years with opioid prescription | Beers (2019) | All-cause mortality within 90 days | 3.76 / 1.72-8.23 | yes / n.a. |
|  | 01.2011- | case-control study (in | (58.5%) |  | claim during follow-up period (1, 1.5, 2, |  |  |  |  |
|  | 05.2017 | underlying cohort of |  |  | 3, and 4 months) and none before and |  |  |  |  |
|  |  | opioid users) |  |  | without malignant tumour diagnosis |  |  |  |  |

Abbrevations: AE = Adverse Events, ADE = Adverse Drug Event, ED = Emergency Department, ICU = Intensive Care Unit, IQR = Interquartile Range, SD = Standard Deviation.

*England, Estonia, Finland, France, Germany, the Netherlands, Spain and Sweden

## S6- Calculations of Variables

With the aid of [Table 13](#_bookmark61) below the Odds Ratio (OR) was calculated using [Equation 1.](#_bookmark62) The 95% Confidence Interval (CI) was calculated by first calculating the Standard Deviation (SD) by application of [Equation 2](#_bookmark63) and in the final step [Equation 3.](#_bookmark64)

Table S13: Table for calculation of Odds Ratio (OR).

|  | **Exposure** | **No Exposure** | **Total** |
| --- | --- | --- | --- |
| **Outcome** | a | c | a + c |
| **No Outcome** | b | d | b + d |
| **Total** | a + b | c + d |  |

*OR* =

*a ∗ d*

*b ∗ d*

(1)

*SD* = r 1 + 1 + 1 + 1

(2)

*a b c d*

*CI* = *eln*(*OR*)*±*1*.*96*∗SD* (3)

The variance (Var) was calculated with [Equation 4](#_bookmark65) and the study weights with [Equation 5.](#_bookmark66)

*V ar* = *SD*^2^ = *τ* ^2^ (4)

1 1

*Study weight* = *V ar* = *SD*2 (5)

## S7 Forest Plots of Additional Analyses

### Outcome Rehospitalisation:


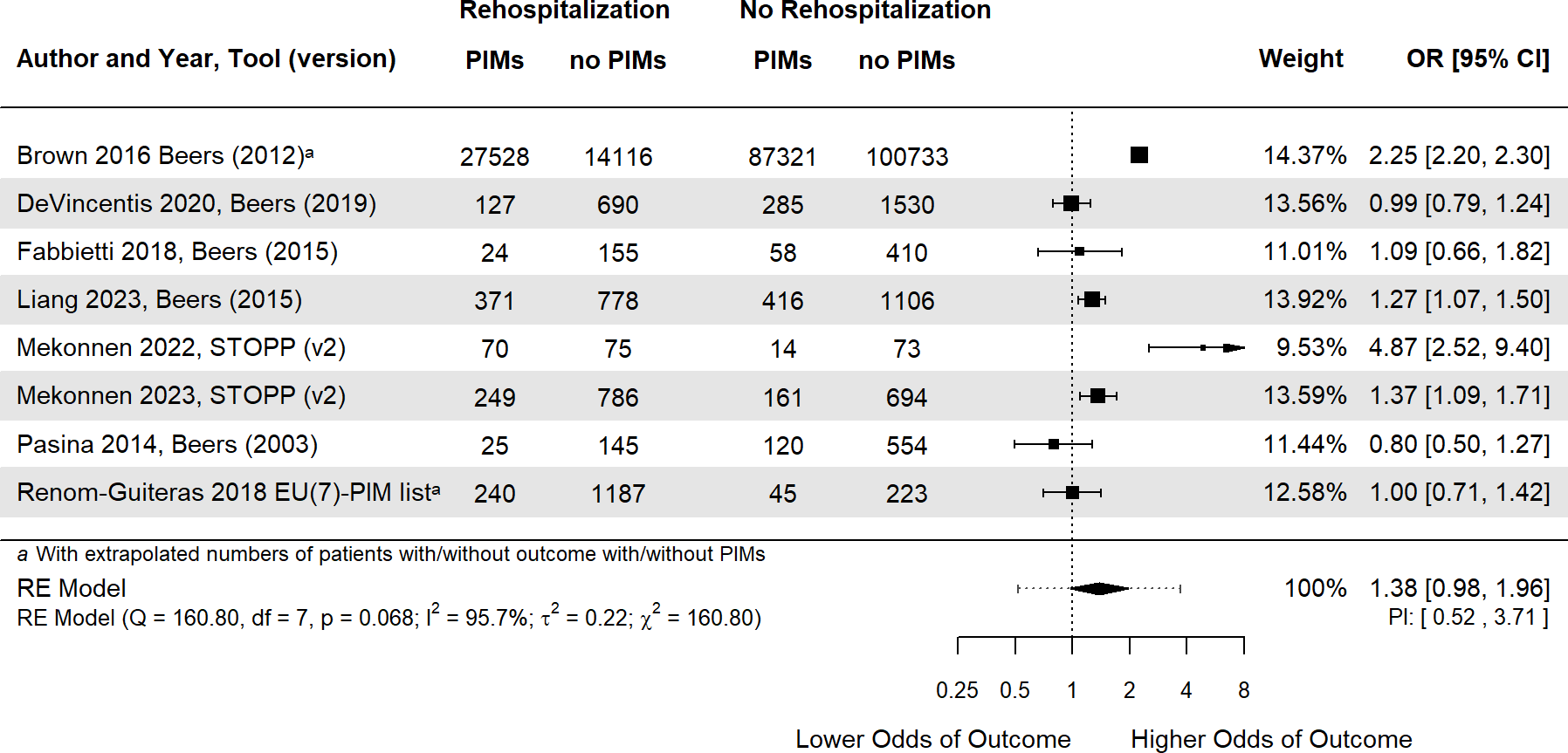


Figure S4: Forest plot of the analysis of the association between PIMs and rehospitalisation within three months. Second ORs of Brown 2016, Fabbietti 2018, DeVincentis 2020, and Pasina 2014.


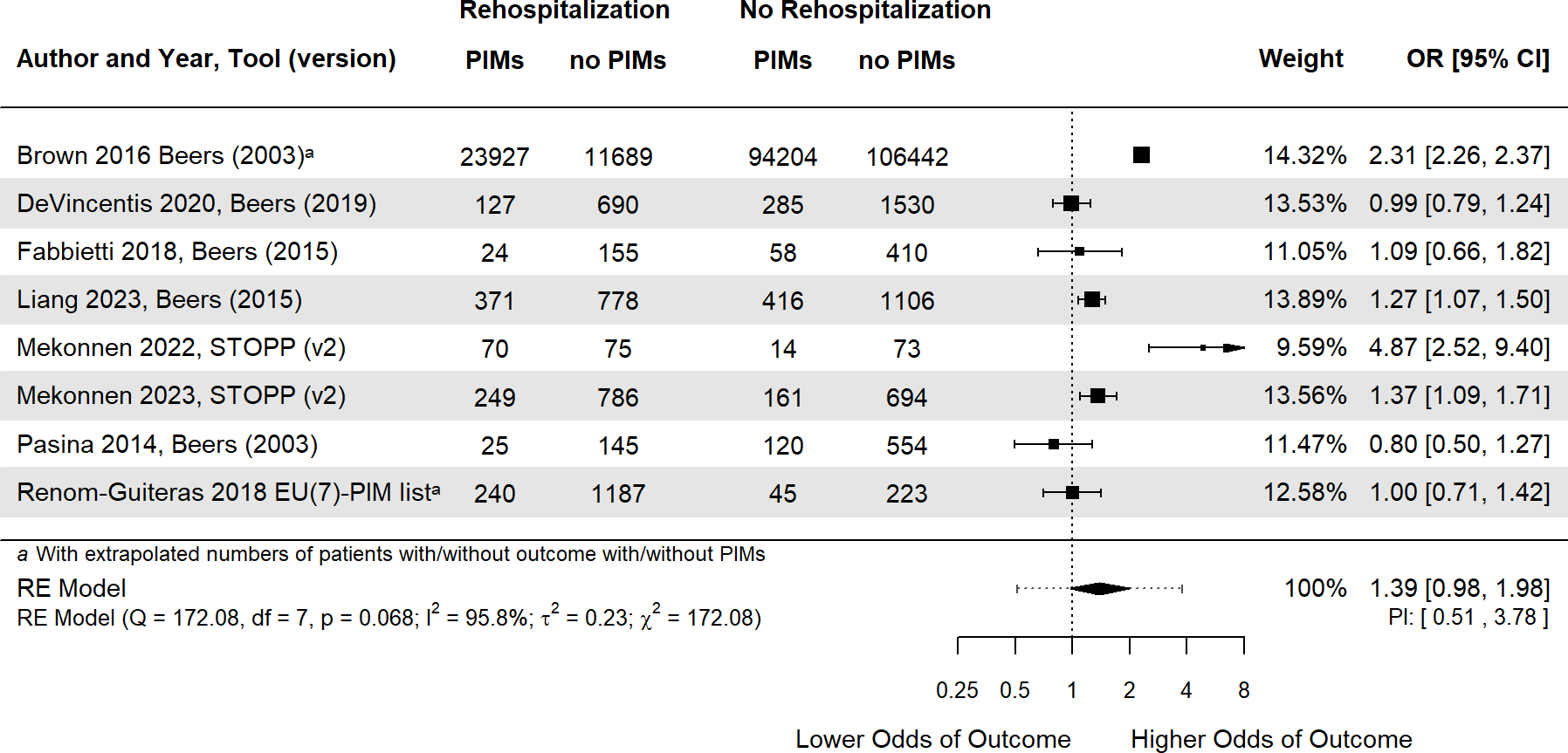


Figure S5: Forest plot of the analysis of the association between PIMs and rehospitalisation within three months. Second ORs of Fabbietti 2018, DeVincentis 2020, and Pasina 2014, third OR of Brown 2016.


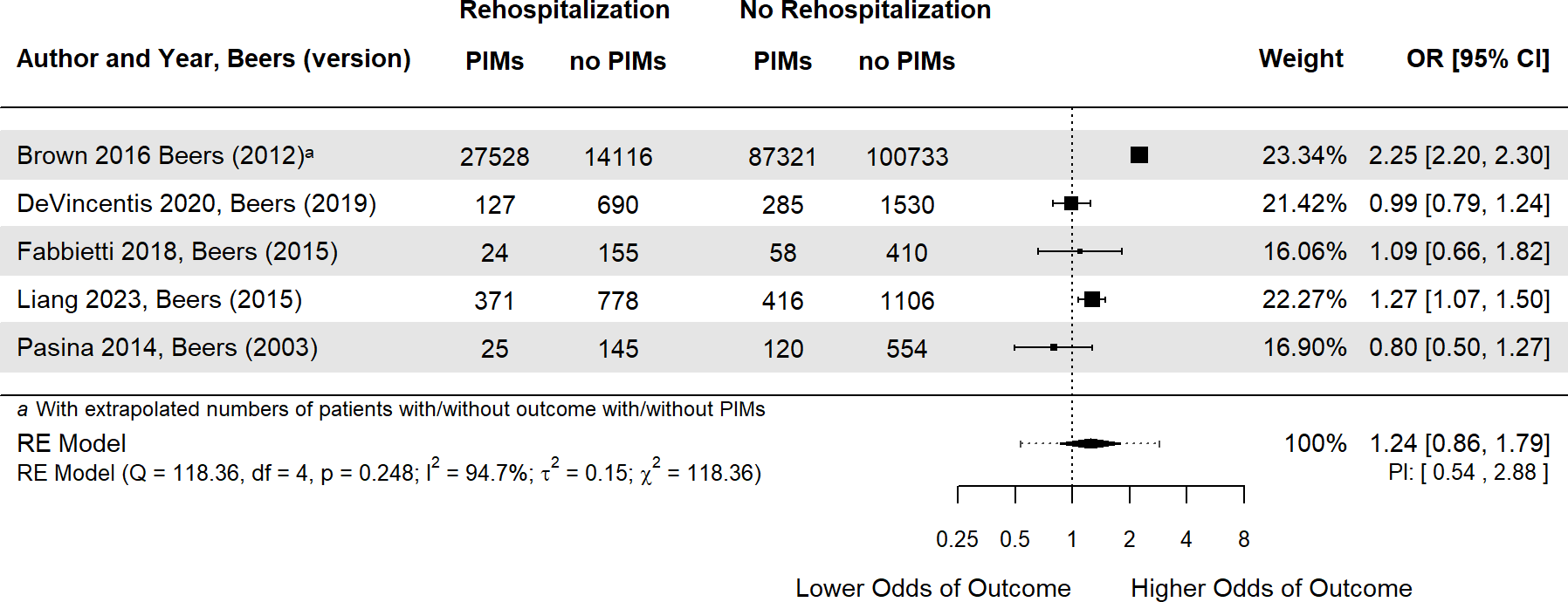


Figure S6: Forest plot of the analysis of the association between Beers PIMs and rehospitalisation within three months. Second Beers OR of Brown 2016.

### Outcome Death:


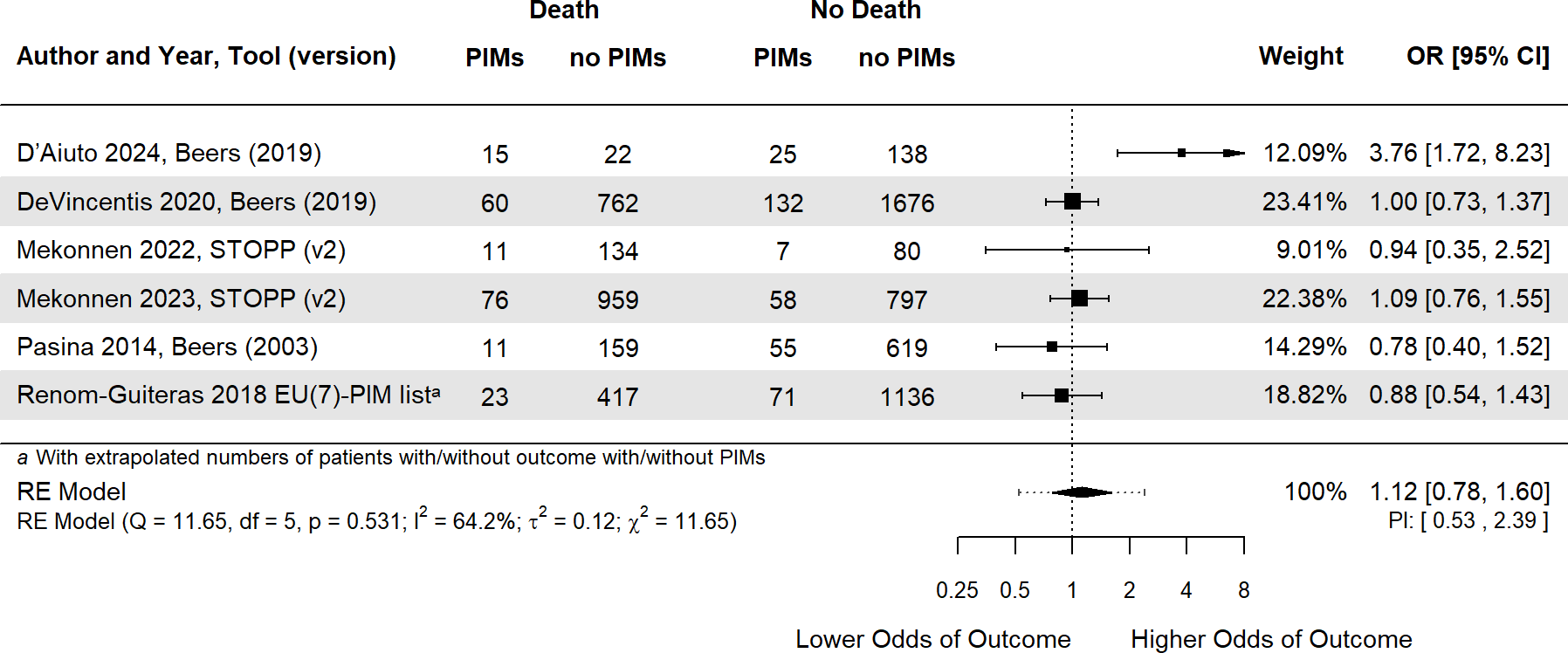


Figure S7: Forest plot of the analysis of the association between Beers PIMs and rehospitalisation within three months. Second ORs of DeVincentis 2020 and Pasina 2014.

## S8 Sensitivity Analysis

### Outcome Rehospitalisation:


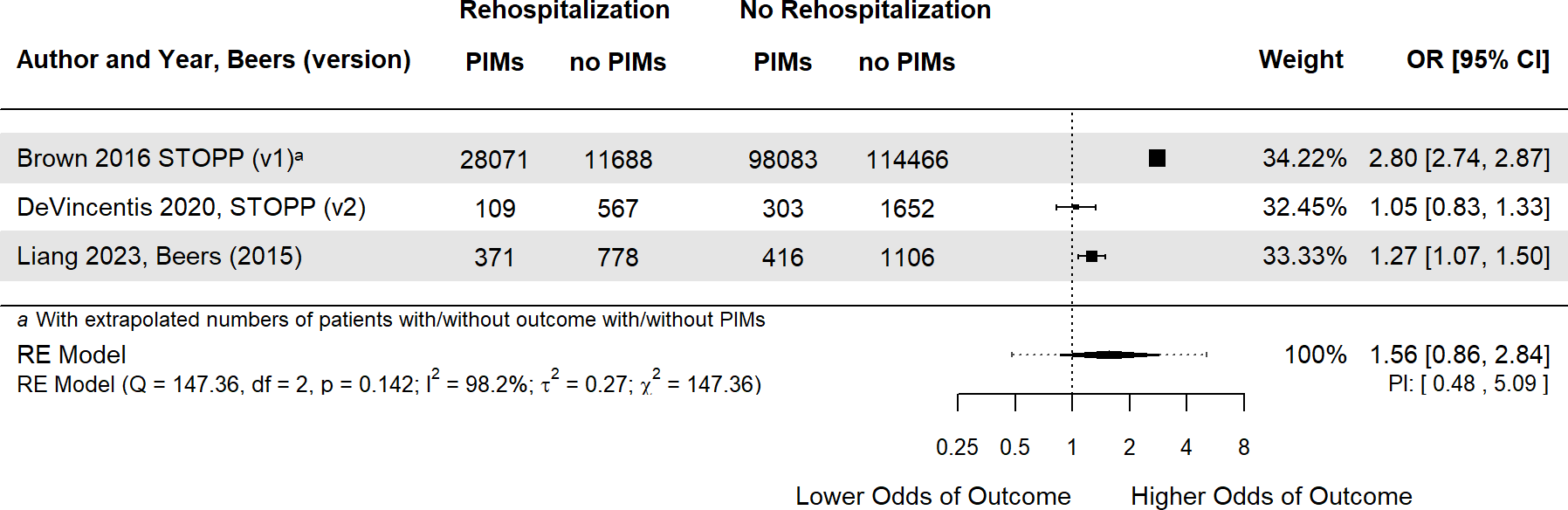


Figure S8: Forest plot of the analysis of the association between Beers PIMs and rehospitalisation within three months without studies with serious RoB.

### Outcome Death:


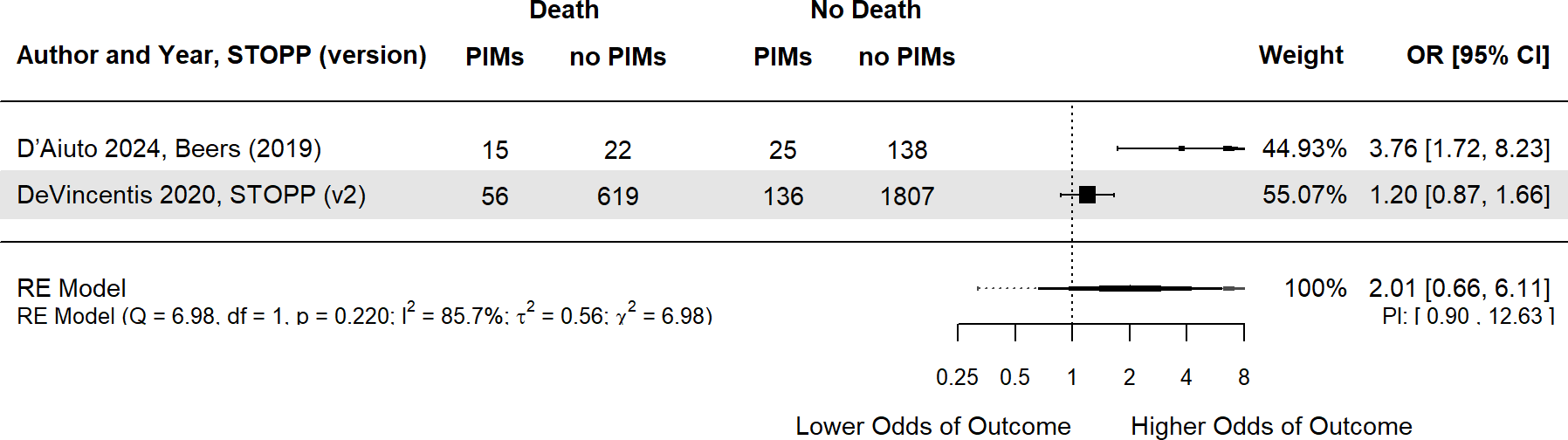


Figure S9: Forest plot of the analysis of the association between Beers PIMs and death within three months without studies with serious RoB.

## S9 Funnel Plots for Publication Bias Assessment

### Outcome Rehospitalisation:


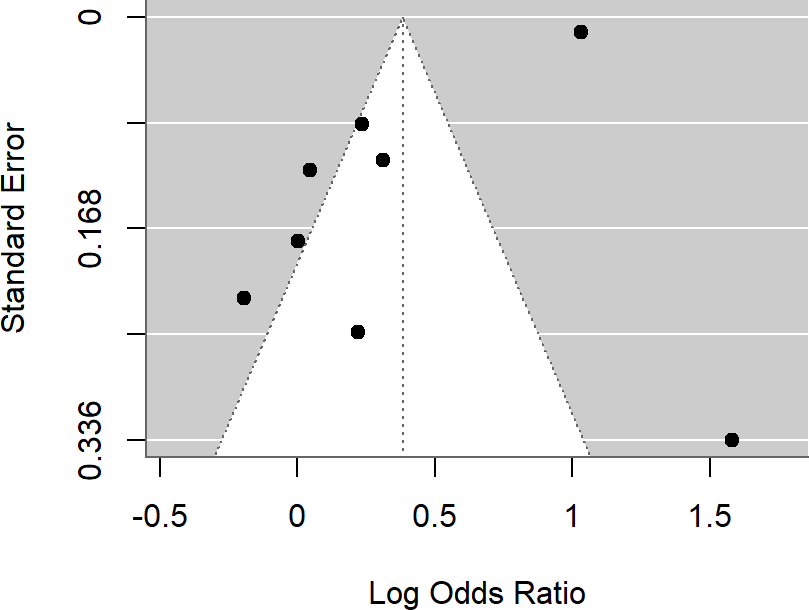


Figure S10: Funnel plot of all included studies for rehospitalisation within three months.


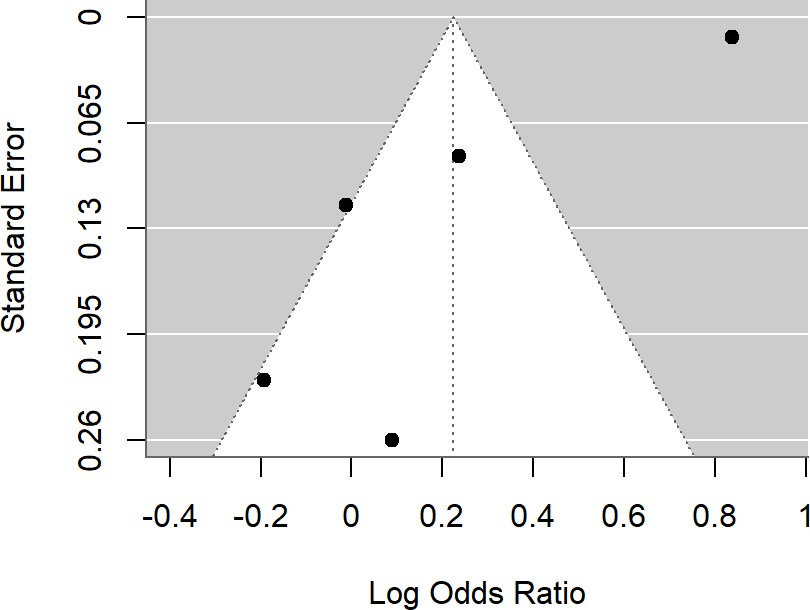


Figure S11: Funnel plot of included studies applying Beers criteria for rehospitalisation within three months.


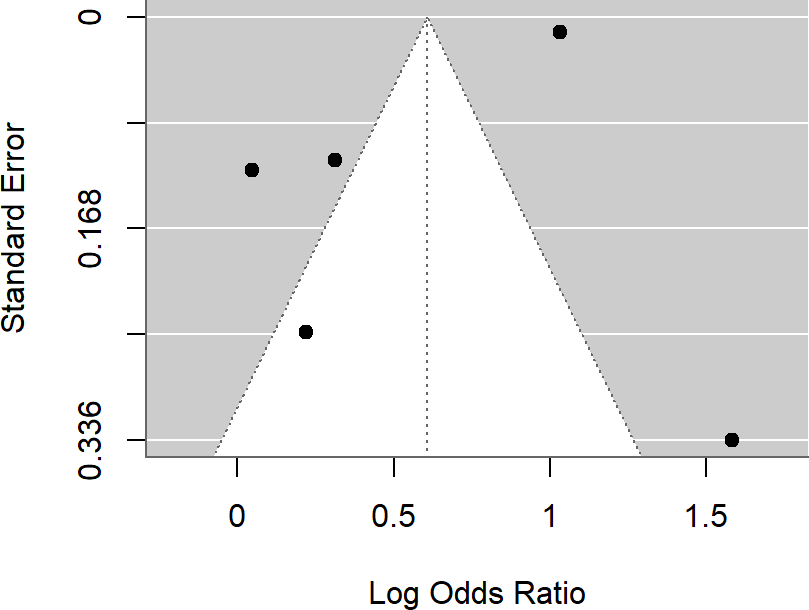


Figure S12: Funnel plot of included studies applying STOPP criteria for rehospitalisation within three months.


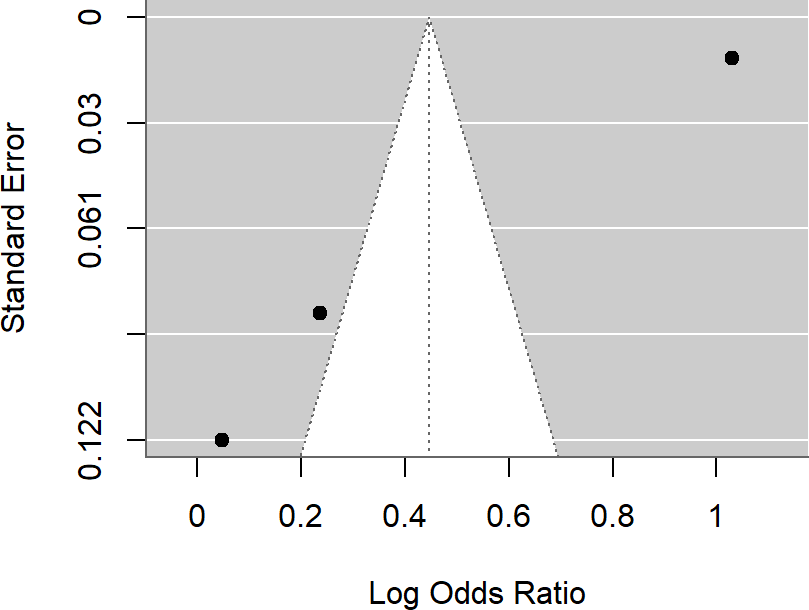


Figure S13: Funnel plot of the included studies with moderate RoB for rehospitalisation within three months.

### Outcome Death:


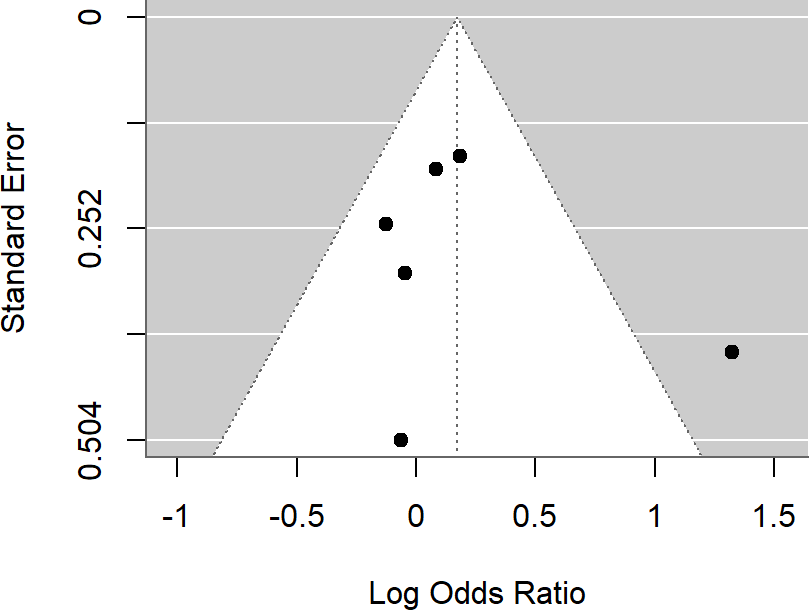


Figure S14: Funnel plot of all included studies for death within three months.


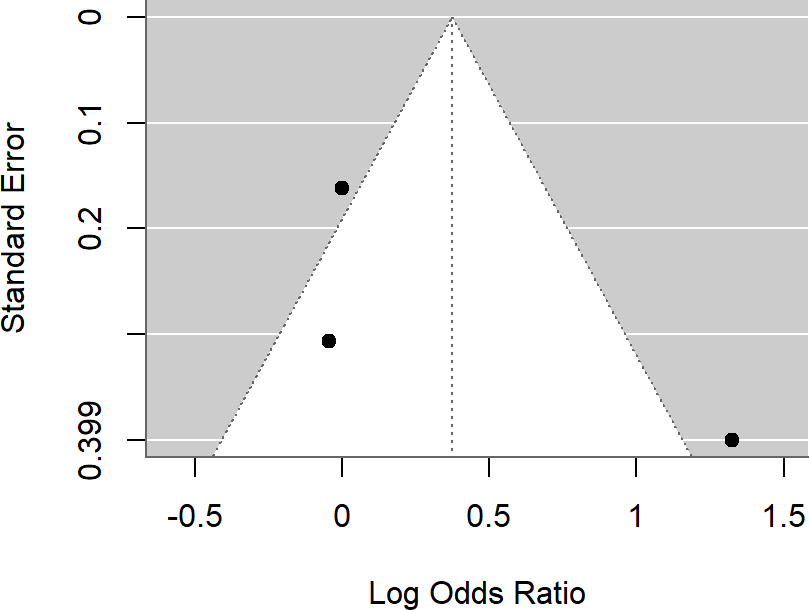


Figure S15: Funnel plot of included studies applying Beers criteria for death within three months.


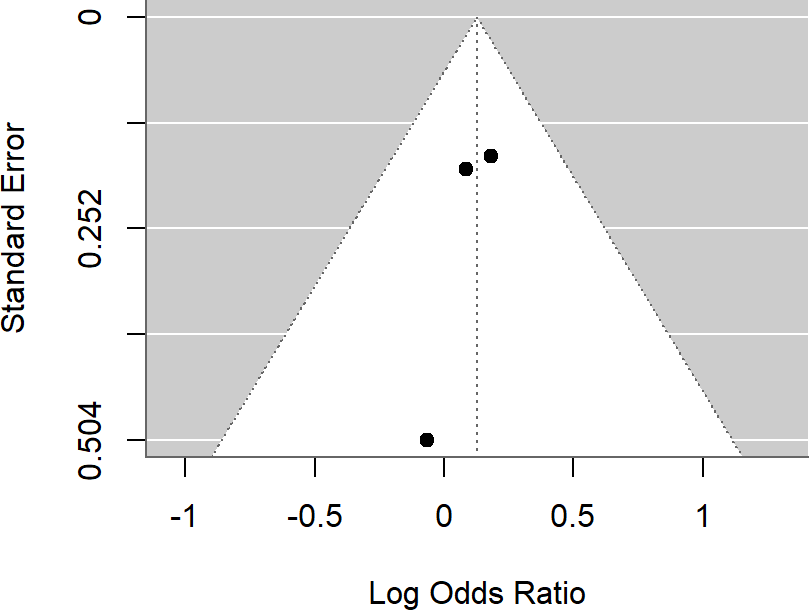


Figure S16: Funnel plot of included studies applying STOPP criteria for death within three months.


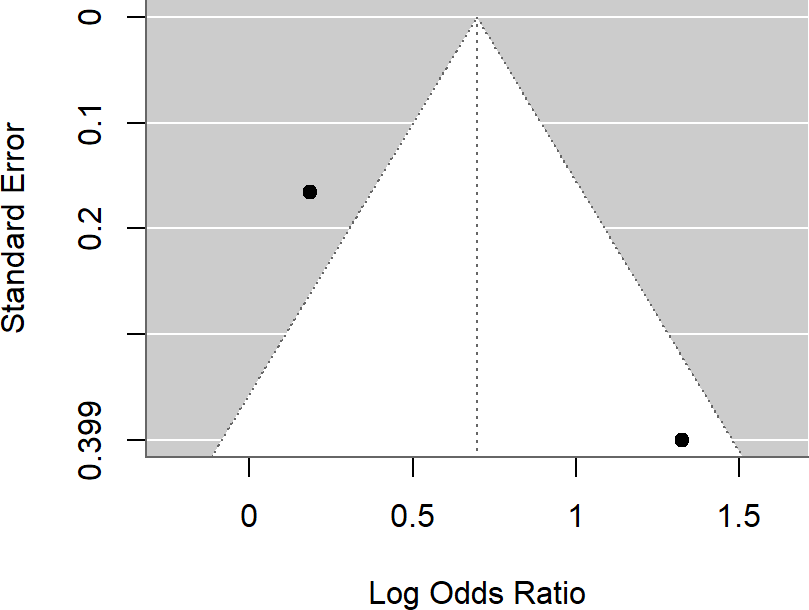


Figure S17: Funnel plot of the included studies with moderate RoB for death within three months.
